# Performance Analysis of Real-Time GPS/Galileo Precise Point Positioning Integrated with Inertial Navigation System

**DOI:** 10.3390/s23052396

**Published:** 2023-02-21

**Authors:** Lei Zhao, Paul Blunt, Lei Yang, Sean Ince

**Affiliations:** Nottingham Geospatial Institute, The University of Nottingham, Nottingham NG7 2TU, UK

**Keywords:** GPS/Galileo, PPP/INS integration, real time, uncombined bias correction, ambiguity resolution

## Abstract

The integration of global navigation satellite system (GNSS) precise point positioning (PPP) and inertial navigation system (INS) is widely used in navigation for its robustness and resilience, especially in case of GNSS signal blockage. With GNSS modernization, a variety of PPP models have been developed and studied, which has also led to various PPP/INS integration methods. In this study, we investigated the performance of a real-time GPS/Galileo zero-difference ionosphere-free (IF) PPP/INS integration with the application of uncombined bias products. This uncombined bias correction was independent of PPP modeling on the user side and also enabled carrier phase ambiguity resolution (AR). CNES (Centre National d’Etudes Spatiales) real-time orbit, clock, and uncombined bias products were used. Six positioning modes were evaluated, including PPP, PPP/INS loosely coupled integration (LCI), PPP/INS tightly coupled integration (TCI), and three of these with uncombined bias correction through a train positioning test in an open sky environment and two van positioning tests at a complex road and city center. All of the tests used a tactical-grade inertial measurement unit (IMU). In the train test, we found that ambiguity-float PPP had almost identical performance with LCI and TCI, which reached an accuracy of 8.5, 5.7, and 4.9 cm in the north (N), east (E) and up (U) direction, respectively. After AR, significant improvements on the east error component were achieved, which were 47%, 40%, and 38% for PPP-AR, PPP-AR/INS LCI, and PPP-AR/INS TCI, respectively. In the van tests, frequent signal interruptions due to bridges, vegetation, and city canyons make the IF AR difficult. TCI achieved the highest accuracies, which were 32, 29, and 41 cm for the N/E/U component, respectively, and also effectively eliminated the solution re-convergence in PPP.

## 1. Introduction

GNSS Precise Point Positioning (PPP) emerged in the late 1990s and was originally for analysing GPS data from large networks [1]. It was well-known for its flexibility of single receiver and capability of centimeter-level high-accuracy positioning. However, PPP requires a convergence process to obtain such high accuracy, which is typically approximately 30 min, but it can vary from minutes to hours, depending on the specific observational conditions [2].

The carrier-phase ambiguity resolution in PPP was then exploited to accelerate the convergence time and improve the positioning accuracy for the GPS legacy L1/L2 frequencies. This technique required the computation of satellite phase biases from a network of stations, which were then disseminated as a correction stream to a user side to enable PPP ambiguity resolution (AR). The satellite phase biases were usually represented in a combined form: the wide-lane (WL) un-calibrated phase delays (UPDs), and the narrow-lane (NL) UPD [3] or the WL satellite biases (WSB) and the ’integer’ phase clocks [4] or the ’decoupled’ clock model [5]. This bias form assumed that the phase ionosphere-free (IF) combination was used at the user end, and after having this bias had been applied, the NL ambiguity with a wavelength around 10 cm could be resolved.

As GNSS evolved, including the modernization of GPS and GLONASS and the newly deployed Galileo and BeiDou, the aforementioned dual-frequency PPP AR methods using combined bias products were extended to other constellations [6,7,8,9]. In the GPS triple-frequency case, the inter-frequency clock bias (IFCB) between the L1/L2 and the L1/L5 clock offset varied with peak-to-peak amplitudes of 10–40 cm [10], and therefore, many studies investigated the estimation of IFCB for its compensation in GPS triple-frequency (TF) PPP [8,11,12] and also demonstrated the GPS TF AR accordingly [13,14]. For Galileo E1/E5a/E5b PPP, it was found that the magnitude of the time-varying IFCB was negligible, and the ambiguity-fixed solutions have also been presented in many studies [13,14,15]. In [16], a Galileo five-frequency PPP with AR by using pairs of classical ionosphere-free combinations on different frequencies was demonstrated.

However, the combined bias formulations were inconvenient when extended for multi-frequency conditions since there were many more possible combinations [17,18], so a new uncombined phase bias representation was proposed in [17,18], which used the same adding convention as the existing RTCM (Radio Technical Commission for Maritime Services) standard for the code biases. This uncombined bias formulation was extended for multi-frequency code and phase observations easily, and it also considered the IFCB effect of GPS Block IIF satellites [17]. Most importantly, the phase ambiguity or its linear combination could still preserve the integer property [19]. Currently, many other studies have also demonstrated the estimation of the uncombined bias products for PPP AR [20,21,22,23].

The inertial navigation system (INS) is an autonomous system that does not require measurements for external signals. It provides high-accuracy short-term position, velocity, and attitude at a high data rate. The integration of GNSS (e.g., PPP) and INS uses the advantages of each system and has good resilience in navigation. The loosely coupled integration (LCI) of GNSS and INS has been used in the domain of solutions [24,25], and the the GNSS receiver was treated as a ’black box’. However, if there were a GNSS signal outage, the inertial sensor would immediately fail to obtain the calibration from GNSS and, thus, output drifted solutions that may not be acceptable. The the coupled integration (TCI) is in the GNSS measurements domain. A TCI structure limits the problems due to signal blockage and benefits from GNSS measurement updates, even when there are less than four satellite. It is then possible for a TCI system to retain high positioning accuracy in harsh GNSS reception environments. In terms of PPP/INS integration, the development of the PPP technique has led to a great variety of PPP/INS TCI models. In [26], the model of a tightly coupled GPS dual-frequency ionosphere-free (IF) PPP and INS integration was first proposed. It was an undifferenced PPP model, as in [27,28] and the phase ambiguities were real values. In [29], the GPS PPP/micro-electro-mechanical system (MEMS) IMU TCI was investigated, and it was found that the between-satellite single-difference (BSSD) IF model performed better, in general, than the undifferenced model. In [30], the results of the TCI of GPS/BeiDou PPP and four different grades of INS were evaluated. Their model was an ionosphere-estimated model and the a priori constraints on the slant ionospheric delays were from the IGS global ionosphere maps (GIM) products. In [31], the tightly coupled GPS/GLONASS/Galileo/BeiDou PPP/MEMS IMU integration was assessed with a dual-frequency IF model for PPP.

As PPP with (AR) has been increasingly studied, PPP/INS integration with AR has also been demonstrated. In [32,33], the GPS PPP/INS TCI with AR was studied. The between-satellite single-difference PPP model is used and the CNES WSB and ’integer’ phase clock products were applied for phase-bias correction. In [34], they studied the ambiguity-fixed GPS PPP/INS TCI. The UPD products were used for AR, and their model was also single-differenced and ionosphere-free. Recently, undifferenced and uncombined PPP models using external precise atmospheric information for integrated navigation were also demonstrated [35,36,37,38], which was also referred as PPP-RTK.

Based on the above review of PPP and PPP/INS methods, the application of uncombined bias products to real-time zero-difference PPP/INS integration has not been comprehensively studied. This uncombined bias correction has been independent of PPP models on the user side and also enabled the resolution of phase ambiguity or its linear combinations. In this study, we applied the real-time uncombined bias products to an IF PPP/INS integration and evaluated its positioning performance, including LCI and TCI, through three real navigation tests.

This study is organised as follows: Section 2 gives the detailed mathematical model for PPP/INS integration with uncombined bias correction; the integrated results are presented in Section 3, followed by a brief discussion in Section 4. Finally, the results are summarised in Section 5.

## 2. Methods

The uncombined code and phase bias products were designed to add the raw code and phase measurements, enabling phase ambiguity resolution of the arbitrary linear combination on the user’s side. This section presents the GPS/Galileo ionosphere-free PPP AR model using uncombined bias products first; then its integration with INS including LCI and TCI follow.

### 2.1. GPS/Galileo Dual-Frequency Ionosphere-Free Observational Model Using Uncombined Biases

#### 2.1.1. Uncombined Formulation

Based on CNES uncombined bias formulations [19,39], the basic GPS code and phase observables from satellite *s* tracked at receiver *r* were modelled as:

(1) $$\begin{array}{cc} \; & P_{1}=\rho +h_{r}-h^{s}+b_{P_{1},r}-b_{P_{1}}^{s}+I+T+\xi_{P_{1}}\\ \; & P_{2}=\rho +h_{r}-h^{s}+b_{P_{2},r}-b_{P_{2}}^{s}+\gamma_{2}I+T+\xi_{P_{2}}\\ \; & \lambda_{1}L_{1}=\rho +h_{r}-h^{s}+b_{L_{1},r}-b_{L_{1}}^{s}-I+T+\lambda_{1}W+\lambda_{1}N_{1}+\xi_{L_{1}}\\ \; & \lambda_{2}L_{2}=\rho +h_{r}-h^{s}+b_{L_{2},r}-b_{L_{2}}^{s}-\gamma_{2}I+T+\lambda_{2}W+\lambda_{2}N_{2}+\xi_{L_{2}} \end{array}$$

where:

Variables *P* and *L* denote the code (in meter) and phase (in cycle) measurements, respectively.

The variable ρ is the geometric propagation distance of the GPS radio wave between *s* and *r* antenna phase center including PCO (phase centre offset) corrections on different frequencies($f_{1},f_{2}$).

Variables $h_{r}$ and $h^{s}$ are the receiver and satellite clock offsets, respectively.

*I* is the slant ionospheric delay at $f_{1}$ for code and is inversely corrected for phase. $\gamma_{2}=f_{1}^{2}/f_{2}^{2}$.

The variable *T* is the slant tropospheric delay.

Furthermore, $\lambda_{i}=c/f_{i}\left(i=1,2\right)$ is the signal wavelength at frequency $f_{i}$ with *c* the speed of light.

The variable *W* is the phase wind-up effect (cycle) which is caused by the relative orientation change between transmitter and receiver antenna for the right circularly polarized GNSS signals [40].

The variable *N* is the carrier phase ambiguity and has the integer property (cycle) by definition.

Variables $b_{r}$ and $b^{s}$ denote the signal hardware delays from receiver and satellite, respectively. These delays are also dependent on the specific observables, i.e., $b_{P_{1},r}$, $b_{P_{1}}^{s}$, $b_{L_{1},r}$ and $b_{L_{1}}^{s}$.

The variable ξ groups the unmodelled errors, such as noise and multipath (m).

The geometric distance between *r* and *s* was computed as:

(2) $$\rho =\sqrt{{\left(X^{s}-X_{r}\right)}^{2}+{\left(Y^{s}-Y_{r}\right)}^{2}+{\left(Z^{s}-Z_{r}\right)}^{2}}$$

where $\left(X^{s},Y^{s},Z^{s}\right)$ and $\left(X_{r},Y_{r},Z_{r}\right)$ are the satellite *s* and receiver *r* coordinates, respectively, in a Earth-centered-Earth-fixed (ECEF) frame.The satellite coordinates were obtained from the satellite ephemerides, such as the CNES precise orbit products, and this equation had to be linearised before estimating $\left(X_{r},Y_{r},Z_{r}\right)$.

After applying CNES real-time precise satellite clock products and also the uncombined bias products, the terms $h^{s}$, $b_{P}^{s}$ and $b_{L}^{s}$ were eliminated from the above equations. As a consequence, one receiver clock per observable had to be re-parameterised on the user’s side. Alternatively, a common receiver clock offset with additional receiver clock biases was defined in these equations as:

(3) $$\begin{array}{cc} \; & P_{1}=\rho +dt^{G}+I+T+\xi_{P_{1}}\\ \; & P_{2}=\rho +dt^{G}+b_{P_{2}}+\gamma_{2}I+T+\xi_{P_{2}}\\ \; & \lambda_{1}L_{1}=\rho +dt^{G}+b_{L_{1}}-I+T+\lambda_{1}W+\lambda_{1}N_{1}+\xi_{L_{1}}\\ \; & \lambda_{1}L_{2}=\rho +dt^{G}+b_{L_{2}}-\gamma_{2}I+T+\lambda_{2}W+\lambda_{2}N_{2}+\xi_{L_{2}} \end{array}$$

where $dt^{G}=h_{r}+b_{P_{1},r}$ is the common GPS receiver clock offset. $b_{P_{2}}=b_{P_{2},r}-b_{P_{1},r}$, $b_{L_{1}}=b_{L_{1},r}-b_{P_{1},r}$, $b_{L_{2}}=b_{L_{2},r}-b_{P_{1},r}$.

#### 2.1.2. Combined Formulation

Making the standard ionosphere-free (*IF*) combination on the two frequencies, the *IF* code and phase observation equations were, as follows:

(4) $$\begin{array}{cc} \; & P_{IF}=\alpha P_{1}+\beta P_{2}=\rho +dt_{P}^{G}+T+\xi_{P_{IF}}\\ \; & \lambda_{IF}L_{IF}=\alpha \lambda_{1}L_{1}+\beta \lambda_{2}L_{2}=\rho +dt_{L}^{G}+T+\lambda_{IF}W+B_{IF}+\xi_{L_{IF}} \end{array}$$

where $\alpha =\frac{f_{1}^{2}}{f_{1}^{2}-f_{2}^{2}},\beta =1-\alpha$, $dt_{P}^{G}=dt^{G}+\beta b_{P_{2}}$; $dt_{L}^{G}=dt^{G}+\left(\alpha b_{L_{1}}+\beta b_{L_{2}}\right)$; $B_{IF}=\lambda_{IF}N_{IF}=\alpha \lambda_{1}N_{1}+\beta \lambda_{2}N_{2}$, which can be decomposed as widelane (*WL*) ambiguity $N_{WL}$ and narrowlane (*NL*) ambiguity $N_{1}$:

(5) $$\begin{array}{c} B_{IF}=\lambda_{IF}N_{IF}=\lambda_{NL}\left(N_{1}+\frac{\lambda_{WL}}{\lambda_{2}}N_{WL}\right) \end{array}$$

where $\lambda_{NL}=\frac{c}{f_{1}+f_{2}}\approx 11\;\mathrm{cm}$ is the *NL* wavelength; $\lambda_{WL}=\frac{c}{f_{1}-f_{2}}\approx 86\;\mathrm{cm}$ is the *WL* wavelength.

The WL ambiguity $N_{WL}$ was resolved in advance using the classical Melbourne–Wübbena (*MW*) combination [41,42] with uncombined bias corrections:

(6) $$\begin{array}{cc} MW & =\left(L_{1}-b_{L_{1}}^{p}\right)-\left(L_{2}-b_{L_{2}}^{p}\right)+\alpha_{1}\left(P_{1}-b_{P_{1}}^{p}\right)+\alpha_{2}\left(P_{2}-b_{P_{2}}^{p}\right)\\ \; & =N_{WL}+\mu_{r}^{G}+\xi_{MW} \end{array}$$

where $\alpha_{1}=\frac{\lambda_{1}-\lambda_{2}}{\left(\lambda_{1}+\lambda_{2}\right)\lambda_{1}}$; $\alpha_{2}=\frac{\lambda_{1}-\lambda_{2}}{\left(\lambda_{1}+\lambda_{2}\right)\lambda_{2}}$; $\mu_{r}^{G}=\frac{b_{L_{1}}}{\lambda 1}-\frac{b_{L_{2}}}{\lambda 2}+\alpha_{2}b_{P_{2}}$ is the receiver *WL* bias.

Similarly, Galileo $E_{1}$ and $E_{5a}$ ionosphere-free PPPs using uncombined bias products were modelled as:

(7) $$\begin{array}{cccc} \; & C_{IF_{E_{1}E_{5a}}} & \; & =\alpha_{E_{1}E_{5a}}C_{E_{1}}+\beta_{E_{1}E_{5a}}C_{E_{5a}}=\rho +dt_{C}^{E}+T+\xi_{C_{IF_{E_{1}E_{5a}}}}\\ \; & \lambda_{IF_{E_{1}E_{5a}}}L_{IF_{E_{1}E_{5a}}} & \; & =\alpha_{E_{1}E_{5a}}\lambda_{E_{1}}L_{E_{1}}+\beta_{E_{1}E_{5a}}\lambda_{E_{5a}}L_{E_{5a}}\\ & \; & & =\rho +dt_{L}^{E}+T+\lambda_{IF_{E_{1}E_{5a}}}W+B_{IF_{E_{1}E_{5a}}}+\xi_{L_{IF_{E_{1}E_{5a}}}}\\ \; & B_{IF_{E_{1}E_{5a}}} & \; & =\lambda_{NL_{E_{1}E_{5a}}}\left(N_{E_{1}}+\frac{\lambda_{WL_{E_{1}E_{5a}}}}{\lambda_{E_{5a}}}N_{WL_{E_{1}E_{5a}}}\right)\\ \; & MW_{E_{1}E_{5a}} & \; & =\left(L_{E_{1}}-b_{L_{E_{1}}}^{q}\right)-\left(L_{E_{5a}}-b_{L_{E_{5a}}}^{q}\right)\\ & \; & & +\alpha_{1_{E_{1}E_{5a}}}\left(C_{E_{1}}-b_{C_{E_{1}}}^{q}\right)+\alpha_{2_{E_{1}E_{5a}}}\left(C_{E_{5a}}-b_{C_{E_{5a}}}^{q}\right)\\ \; & \; & & =N_{WL_{E_{1}E_{5a}}}+\mu_{r}^{E}+\xi_{MW_{E_{1}E_{5a}}} \end{array}$$

where $\alpha_{E_{1}E_{5a}}=\frac{E_{1}^{2}}{E_{1}^{2}-E_{5a}^{2}}$; $\beta_{E_{1}E_{5a}}=1-\alpha_{E_{1}E_{5a}}=-\frac{E_{5a}^{2}}{E_{1}^{2}-E_{5a}^{2}}$; $\alpha_{1_{E_{1}E_{5a}}}=\frac{\lambda_{E_{1}}-\lambda_{E_{5a}}}{\left(\lambda_{E_{1}}+\lambda_{E_{5a}}\right)\lambda_{E_{1}}}$; $\alpha_{2_{E_{1}E_{5a}}}=\frac{\lambda_{E_{1}}-\lambda_{E_{5a}}}{\left(\lambda_{E_{1}}+\lambda_{E_{5a}}\right)\lambda_{E_{5a}}}$; $C_{E_{1}}$ and $L_{E_{1}}$ are the Galileo code and phase observation on frequency $E_{1}$, the same for $C_{E_{5}a}$ and $L_{E_{5}a}$; $\mu_{r}^{E}$ is the Galileo receiver WL bias, such as $\mu_{r}^{G}$.

### 2.2. PPP/INS Integration

The discrete extended Kalman filter was used for PPP/INS integration. In the prediction step, the state transition matrix Φ was an exponential series of the dynamic matrix *F* multiplying the time step Δt as $\Phi =e^{F\Delta t}$, and the process noise matrix $Q_{t}$ was approximated as $Q_{t}\approx GQG^{T}\Delta t$ [25], where *G* maps the disturbing forces to the states and *Q* is the spectral density matrix containing IMU sensor noises, standard deviations of sensor biases, and scale factors, (see Equations (10) and (18)). This section provides the detailed expression of F,G,Q and the design matrix *H* in the measurement update step for both the loosely coupled integration and the tightly coupled integration.

#### 2.2.1. Loosely Coupled Integration

For the loosely coupled integration (LCI), the estimated states were expressed as:

(8) $$\begin{array}{c} x={\left[\delta r^{n}\;\delta v^{n}\;\epsilon^{n}\;b_{a}\;b_{g}\;s_{a}\;s_{g}\right]}^{T} \end{array}$$

where $\delta r^{n}$ and $\delta v^{n}$ are the error states of position (φλh) and velocity $\left(v_{N}\;v_{E}\;v_{D}\right)$, respectively; $\delta r^{n}={\left[\delta \varphi \;\delta \lambda \;\delta h\right]}^{T}$, $\delta v^{n}={\left[\delta v_{N}\;\delta v_{E}\;\delta v_{D}\right]}^{T}$; ϵ is attitude error in the form of Euler angle and $\epsilon ={\left[\epsilon_{N}\;\epsilon_{E}\;\epsilon_{D}\right]}^{T}$; *b* stands for biases of IMU sensors; $b_{a}={\left[b_{a_{X}}\;b_{a_{Y}}\;b_{a_{Z}}\right]}^{T}$ is the IMU accelerometer bias vector in the IMU *XYZ* frame; $b_{g}={\left[b_{g_{X}}\;b_{g_{Y}}\;b_{g_{Z}}\right]}^{T}$ is the IMU gyro bias vector in the IMU axis tripod; $s_{a}$ and $s_{g}$ denotes for scale factor of the accelerometer and gyro, which were optional for tactical-grade IMU.

The dynamic matrix was formed as:

(9) $$\begin{array}{c} F=\left[\begin{array}{ccccccc} F_{rr} & F_{rv} & 0_{3\times 3} & 0_{3\times 3} & 0_{3\times 3} & 0_{3\times 3} & 0_{3\times 3}\\ F_{vr} & F_{vv} & \left(f^{n}\times \right) & C_{b}^{n} & 0_{3\times 3} & C_{b}^{n}f^{b} & 0_{3\times 3}\\ F_{er} & F_{ev} & \left(-\omega_{in}^{n}\times \right) & 0_{3\times 3} & C_{b}^{n} & 0_{3\times 3} & C_{b}^{n}\omega_{nb}^{b}\\ 0_{3\times 3} & 0_{3\times 3} & 0_{3\times 3} & \eta_{b_{a}} & 0_{3\times 3} & 0_{3\times 3} & 0_{3\times 3}\\ 0_{3\times 3} & 0_{3\times 3} & 0_{3\times 3} & 0_{3\times 3} & \eta_{b_{g}} & 0_{3\times 3} & 0_{3\times 3}\\ 0_{3\times 3} & 0_{3\times 3} & 0_{3\times 3} & 0_{3\times 3} & 0_{3\times 3} & \eta_{s_{a}} & 0_{3\times 3}\\ 0_{3\times 3} & 0_{3\times 3} & 0_{3\times 3} & 0_{3\times 3} & 0_{3\times 3} & 0_{3\times 3} & \eta_{s_{g}} \end{array}\right] \end{array}$$

where the partial derivatives $F_{rr}$, $F_{rv}$, $F_{vr}$, $F_{vv}$, $F_{er}$ and $F_{ev}$ can be obtained from the INS error dynamics [43]; *f* is the accelerometer measurements; $C_{b}^{n}$ is the rotation matrix from body frame *b* to navigation frame *n*; $\omega_{in}^{n}$ is the rotation vector of navigation frame to inertial frame *i* resolved in the navigation frame; and η is the self-correlation time matrix for IMU sensor bias and scale factor parameters. The spectral density matrix was defined as:

(10) $$\begin{array}{c} Q=diag\{n_{a}\;n_{g}\;\sigma_{b_{a}}^{2}\;\sigma_{b_{g}}^{2}\;\sigma_{s_{a}}^{2}\;\sigma_{s_{g}}^{2}\} \end{array}$$

where $n_{a}$ and $n_{g}$ are accelerometer and gyro noises, respectively, and σ are the corresponding standard deviations. These values were found in the IMU profile [44]. Furthermore, the disturbance mapping matrix was, as follows:

(11) $$\begin{array}{c} G=\left[\begin{array}{cccccc} 0_{3\times 3} & 0_{3\times 3} & 0_{3\times 3} & 0_{3\times 3} & 0_{3\times 3} & 0_{3\times 3}\\ C_{b}^{n} & 0_{3\times 3} & 0_{3\times 3} & 0_{3\times 3} & 0_{3\times 3} & 0_{3\times 3}\\ 0_{3\times 3} & C_{b}^{n} & 0_{3\times 3} & 0_{3\times 3} & 0_{3\times 3} & 0_{3\times 3}\\ 0_{3\times 3} & 0_{3\times 3} & I_{3\times 3} & 0_{3\times 3} & 0_{3\times 3} & 0_{3\times 3}\\ 0_{3\times 3} & 0_{3\times 3} & 0_{3\times 3} & I_{3\times 3} & 0_{3\times 3} & 0_{3\times 3}\\ 0_{3\times 3} & 0_{3\times 3} & 0_{3\times 3} & 0_{3\times 3} & I_{3\times 3} & 0_{3\times 3}\\ 0_{3\times 3} & 0_{3\times 3} & 0_{3\times 3} & 0_{3\times 3} & 0_{3\times 3} & I_{3\times 3} \end{array}\right] \end{array}$$

The measurements in LCI were the position and velocity difference between PPP and INS, which were expressed as:

(12) $$\begin{array}{c} z=\left[\begin{array}{c} S\left(r_{PPP}^{n}-r_{INS}^{n}\right)-C_{b}^{n}l^{b}\\ v_{Doppler}^{n}-v_{INS}^{n}-C_{b}^{n}\left(\omega_{nb}^{b}\times \right)l^{b} \end{array}\right] \end{array}$$

where $l^{b}$ is the lever arm from IMU center to the antenna phase center; *S* is a scale matrix to convert radian to meter:

(13) $$\begin{array}{c} S=\left[\begin{array}{ccc} RN+h & 0 & 0\\ 0 & (RE+h)cos\varphi & 0\\ 0 & 0 & 1 \end{array}\right] \end{array}$$

where RN and RE are the radii of curvature in the meridian and prime vertical. The design matrix was then formed as:

(14) $$\begin{array}{c} H=\left[\begin{array}{ccccccc} S & 0_{3\times 3} & 0_{3\times 3} & 0_{3\times 3} & 0_{3\times 3} & 0_{3\times 3} & 0_{3\times 3}\\ 0_{3\times 3} & I_{3\times 3} & 0_{3\times 3} & 0_{3\times 3} & 0_{3\times 3} & 0_{3\times 3} & 0_{3\times 3} \end{array}\right] \end{array}$$

The measurements noise matrix was expressed as:

(15) $$\begin{array}{c} R=diag\{\sigma_{r_{PPP}^{n}}^{2}\;\sigma_{v_{Doppler}^{n}}^{2}\} \end{array}$$

where the $\sigma_{r_{PPP}^{n}}^{2}\;\sigma_{v_{Doppler}^{n}}^{2}$ can be from the co-variance matrix of parameters estimated in a PPP filter with Doppler measurements.

#### 2.2.2. Tightly Coupled Integration

Assuming *m* GPS satellites (*p*1, …, *p*2) and n Galileo satellites (*q*1, …, *qn*) were tracked for an epoch, the states to be estimated in the tightly coupled integration (TCI) could be:

(16) $$\begin{array}{cc} x=[\; & \delta r^{n}\;\delta v^{n}\;\epsilon^{n}\;b_{a}\;b_{g}\;s_{a}\;s_{g}\;ZTD\;dt_{P}^{G}\;dt_{L}^{G}\;{\dot{dt}}^{G}\;dt_{C}^{E}\;dt_{L}^{E}\;{\dot{dt}}^{E}\;\\ \; & N_{IF}^{p1}\;\cdots \;N_{IF}^{pm}\;N_{IF_{E_{1}E_{5a}}}^{q1}\;\cdots \;N_{IF_{E_{1}E_{5a}}}^{qn}{]}^{T} \end{array}$$

where ZTD is the zenith tropospheric delay. $\dot{dt^{G}}$ and $\dot{dt^{E}}$ are the GPS and Galileo receiver clock bias rate, respectively, which were used for modelling GPS and Galileo Doppler measurements.

The dynamic matrix in this *TCI* case was formed as:

(17) $$\begin{array}{c} F_{TCI}=\left[\begin{array}{cc} F & 0\\ 0 & 0 \end{array}\right] \end{array}$$

where *F* is the same as Equation (9). The spectral density matrix for sensor noises, sensor biases and scale factors, ZTD, and clock and phase ambiguities was formed as:

(18) $$\begin{array}{cc} Q=diag\{ & n_{a}\;n_{g}\;\sigma_{b_{a}}^{2}\;\sigma_{b_{g}}^{2}\;\sigma_{s_{a}}^{2}\;\sigma_{s_{g}}^{2}\;\sigma_{ZTD}^{2}\;\sigma_{dt_{P}^{G}}^{2}\;\sigma_{dt_{L}^{G}}^{2}\;\\ \; & \sigma_{{\dot{dt}}^{G}}^{2}\;\sigma_{{\dot{dt}}^{E}}^{2}\;\sigma_{dt_{C}^{E}}^{2}\;\sigma_{dt_{L}^{E}}^{2}\;0\} \end{array}$$

where the spectral densities of all the ambiguities are set to zero. The disturbance mapping matrix was:

(19) $$\begin{array}{c} G_{TCI}=\left[\begin{array}{cc} G & 0\\ 0 & I \end{array}\right] \end{array}$$

where *G* is equal to Equation (11). Assume the measurement vector was arranged as:

(20) $$\begin{array}{cc} z=[\; & P_{IF}^{p1}\;\lambda_{IF}L_{IF}^{p1}\;\lambda_{L_{1}}D_{L_{1}}^{p1}\;\cdots \;P_{IF}^{pm}\;\lambda_{IF}L_{IF}^{pm}\;\lambda_{L_{1}}D_{L_{1}}^{pm}\;\\ \; & C_{IF_{E_{1}E_{5a}}}^{q1}\;\lambda_{IF_{E_{1}E_{5a}}}L_{IF_{E_{1}E_{5a}}}^{q1}\;\lambda_{E_{1}}D_{E_{1}}^{q1}\;\cdots \;C_{IF_{E_{1}E_{5a}}}^{qn}\;\lambda_{IF_{E_{1}E_{5a}}}L_{IF_{E_{1}E_{5a}}}^{qn}\;\lambda_{E_{1}}D_{E_{1}}^{qn}\;{]}^{T} \end{array}$$

and the design matrix would be:

(21) $$\begin{array}{c} H=\left[\begin{array}{ccccccccccc} H_{r,v}^{p1} & 0 & H_{T}^{p1} & H_{clk}^{p1} & 0 & H_{amb}^{p1} & \cdots & 0 & 0 & \cdots & 0\\ \vdots & \vdots & \vdots & \vdots & \vdots & \vdots & \vdots & \vdots & \vdots & \vdots & \vdots \\ H_{r,v}^{pm} & 0 & H_{T}^{pm} & H_{clk}^{pm} & 0 & 0 & \cdots & H_{amb}^{pm} & 0 & \cdots & 0\\ H_{r,v}^{q1} & 0 & H_{T}^{q1} & 0 & H_{clk}^{q1} & 0 & \cdots & 0 & H_{amb}^{q1} & \cdots & 0\\ \vdots & \vdots & \vdots & \vdots & \vdots & \vdots & \vdots & \vdots & \vdots & \vdots & \vdots \\ H_{r,v}^{qn} & 0 & H_{T}^{qn} & 0 & H_{clk}^{qn} & 0 & \cdots & 0 & 0 & \cdots & H_{amb}^{qn} \end{array}\right] \end{array}$$

where $H_{r,v}$ contains the partial derivatives of code, phase, and Doppler measurements with respect to position and velocity; $H_{T}$ groups the wet mapping functions for ZTD; $H_{clk}^{p}$ and $H_{clk}^{q}$ denote the coefficients for GPS and Galileo clock and clock rates, respectively; and $H_{amb}$ groups the coefficients for ambiguity parameters.

## 3. Results

### 3.1. Experiment Description

#### Test Settings

**Figure 1:**
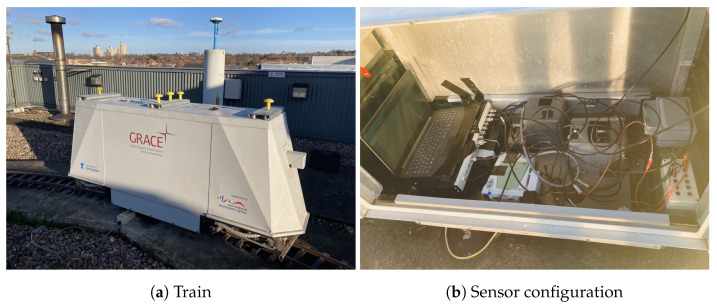
NGI test train.

**Figure 2:**
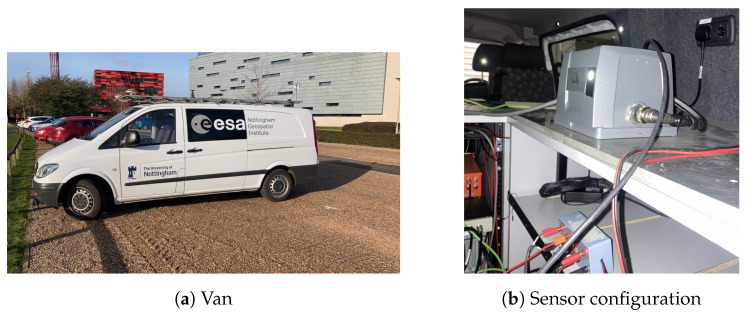
NGI test van.

Three tests were conducted in this study using a test train and a test van at Nottingham Geospatial Institute (NGI), as shown in Figure 1 and Figure 2.

The LEICA GS10 [45] GNSS receiver was used for collecting GNSS measurements. The NovAtel SPAN UIMU-LCI [44] was used for measuring acceleration and rotation, and it is a tactical grade IMU. Table 1 lists the data sampling rate information.

**Figure 3:**
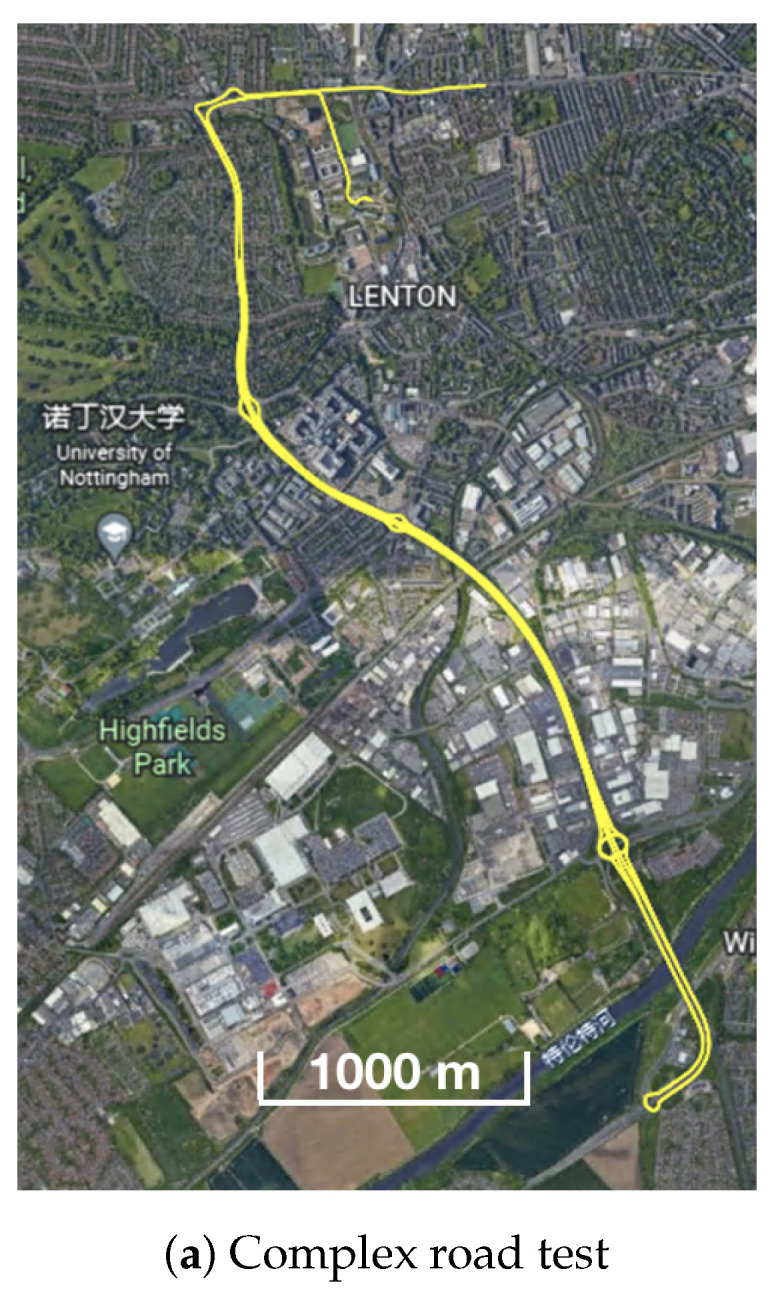

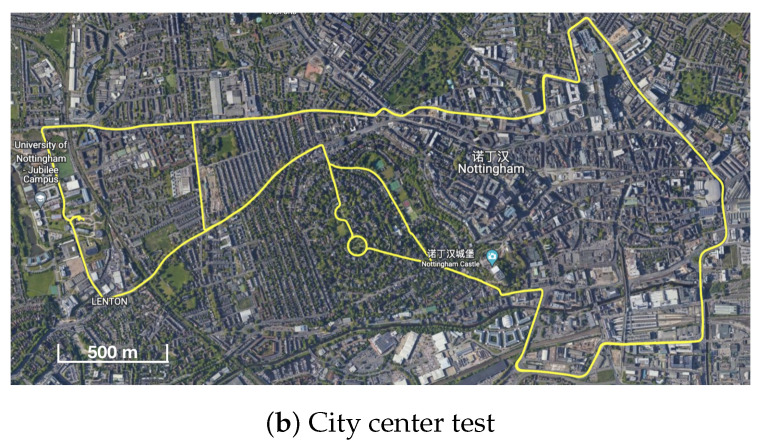
Trajectories of two van positioning tests.

**Figure 4:**
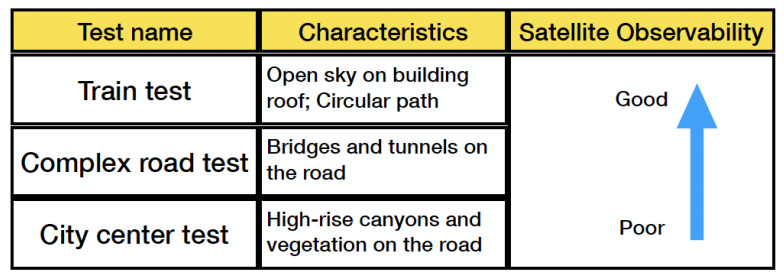
Principle of experimental design.

The train operated on the roof of the Nottingham Geospatial Building (NGB), along a circular path and had good satellite visibility. The van tests were designed on a complex road at the city center, where frequent signal blockage occurred due to bridges, high-rises, and vegetation. Figure 3 shows the van motion trajectories, and Figure 4 summarises the experimental characteristics according to the difficulty level of the GNSS signal reception.

The POINT (Position Orientation and INTegration) software [46,47] was used to compute the real-time GPS/Galileo PPP/INS integration. A PPP settings were, as follows in Table 2:

The commercial software Inertial Explorer was used for reference computations with smoothed GNSS differential positioning and INS TCI modes. Its solution quality or state number indicated different positioning accuracy. Quality 1 had a 3D accuracy of 0–15 cm, Quality 2 of 5–40 cm, and Quality 3 of 20–100 cm [49].

**Figure 5:**
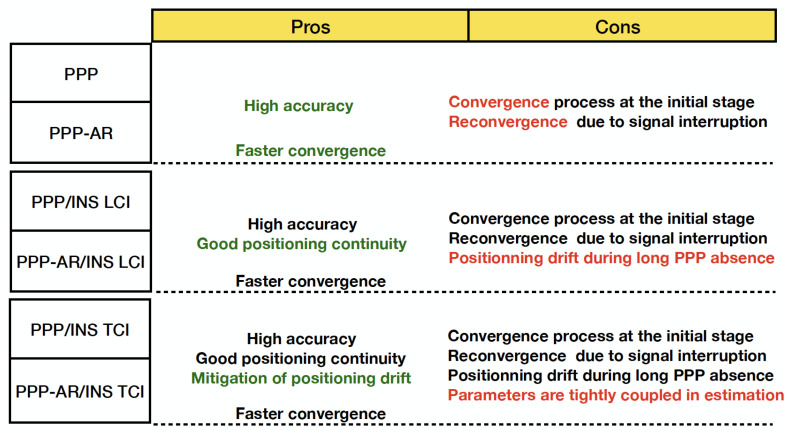
Advantages and disadvantages of six positioning modes.

Six positioning modes are compared in this section, of which the advantages and disadvantages are listed in the chart Figure 5.

### 3.2. Train Positioning Test Results

#### 3.2.1. Positioning Error Evaluation

**Figure 6:**
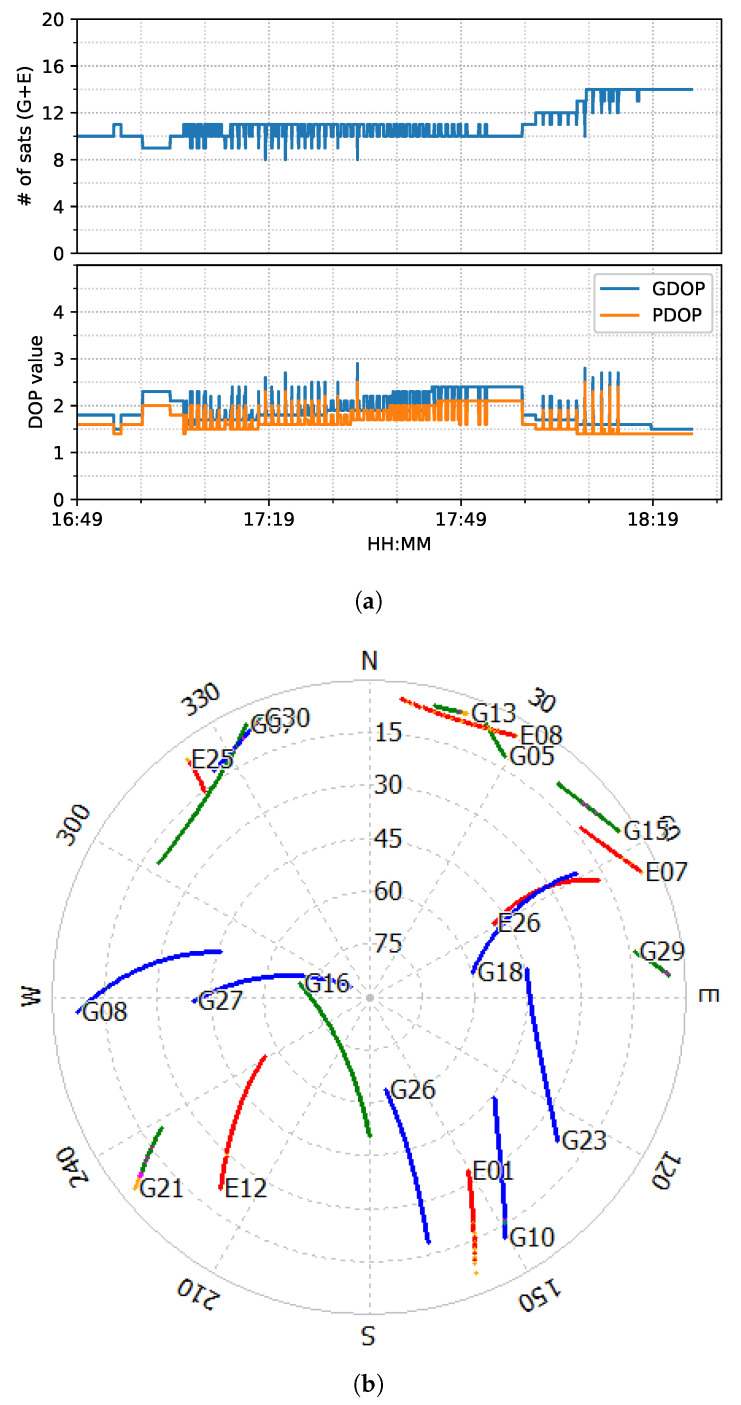
GPS/Galileo satellite information (elevation angle above 10 degrees) of train test on 7 March 2022. (**a**) Number of satellites and DOP values. (**b**) Skyplot. (Different color stands for the availability of different frequencies, generated by RTKLIB [50]).

**Figure 7:**
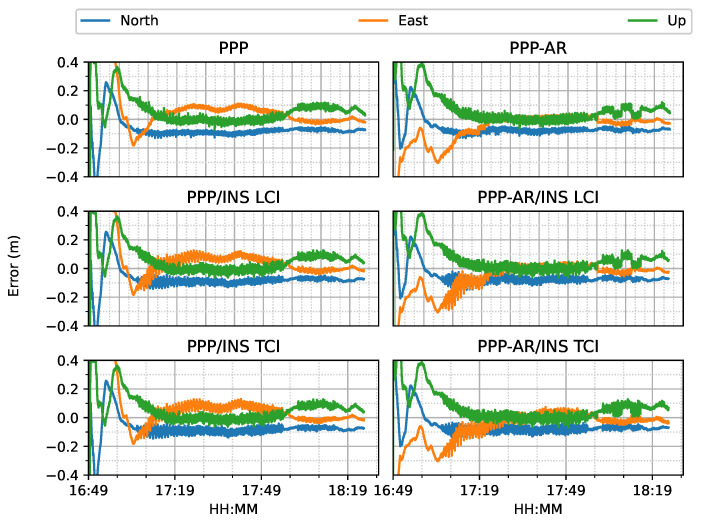
PPP/INS results of the train positioning test on 7 March 2022.

The train test had good satellite observability and low DOP (dilution of precision) values, as shown in Figure 6. It started moving forward at around 17:05 and stopped at around 17:55 for about five minutes. Then, it reversed until 18:15, followed by 10-minute static periods until the end. The positioning results of the six positioning modes are shown in Figure 7.

**Figure 8:**
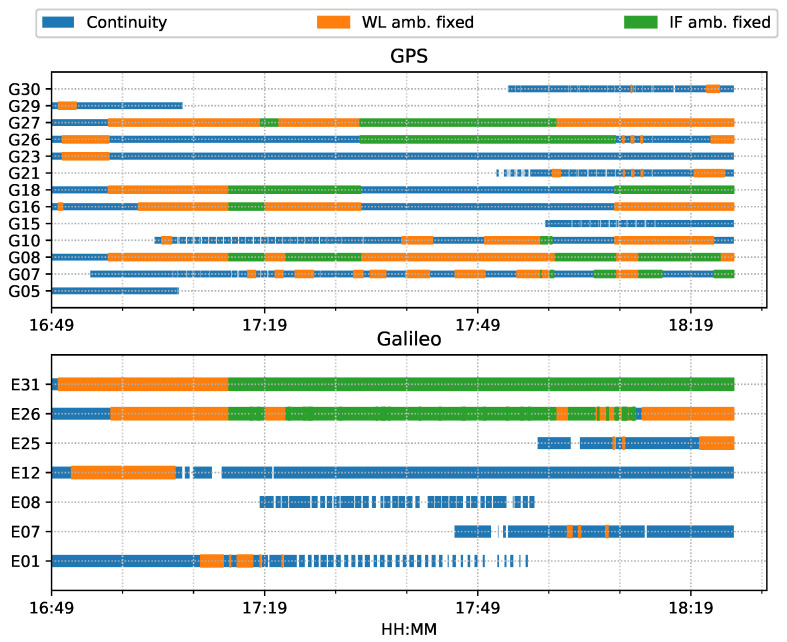
Time span of observed satellites and the fixed ambiguity states of PPP-AR train positioning test on 7 March 2022.

For both ambiguity-float (left column) and ambiguity-fixed (right column) solutions, the integrated results conformed well to the PPP-only solutions. The converged PPP results maintained high positioning accuracy within 10 cm during the test period, mainly because of the optimal observational conditions.With the phase ambiguity resolution (AR), the east component of the three fixed solutions achieved a noticeable improvement, as compared to the related ambiguity-float solutions. However all the solutions still require approximate 25 min to obtain converged solutions. Figure 8 shows the status of the fixed ambiguity for each observed satellite during the train test. More GPS satellites had fixed ionosphere-free (IF) or narrowlane (NL) ambiguities while only two Galileo satellites had fixed IF ambiguities.

**Figure 9:**
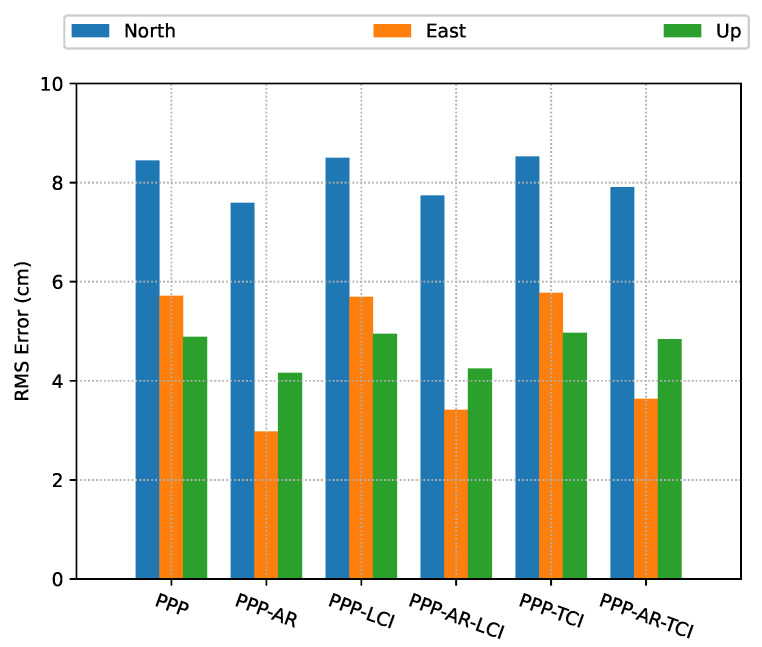
PPP/INS positioning error RMS of the train test on 7 March 2022 with respect to IE RTK solutions.

The positioning error RMS of each mode is given in Figure 9. All the real-time PPP/INS solutions obtained an accuracy below 10 cm in each direction. After AR, all the solutions achieved significant improvements on the east component, which were around 47%, 40%, and 38% for PPP-AR, PPP-AR/INS LCI, and PPP-AR/INS TCI, respectively, as compared to the related ambiguity-float solutions. It should be noted that GNSS data had a lower sampling rate than the IMU, and the integrated solutions were entirely determined by INS before the GNSS and INS time were aligned. Therefore, the PPP-solutions had a smaller data size though PPP-AR slightly outperformed LCI and TCI.

#### 3.2.2. Velocity and Attitude Errors

**Figure 10:**
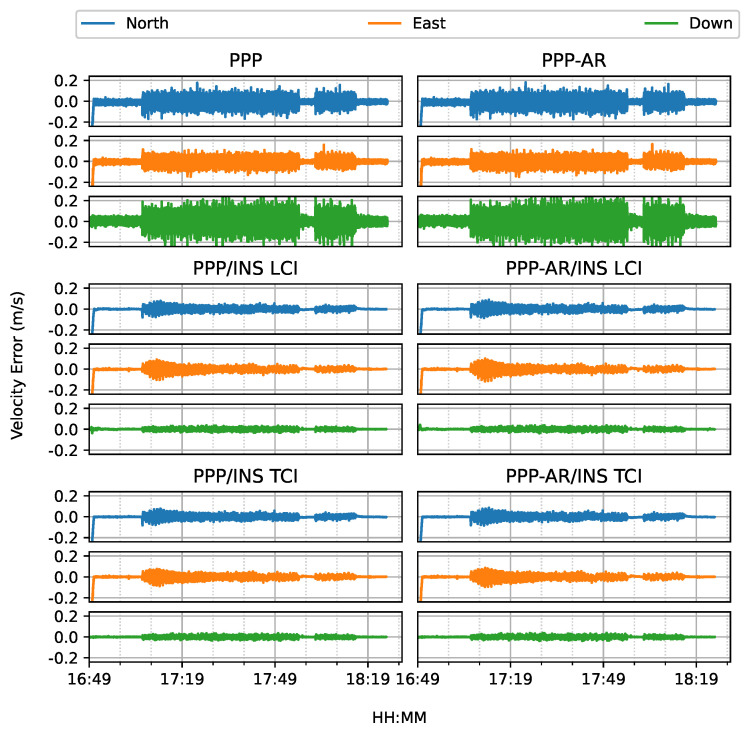
Velocity errors of PPP/INS train positioning test on 7 March 2022.

**Figure 11:**
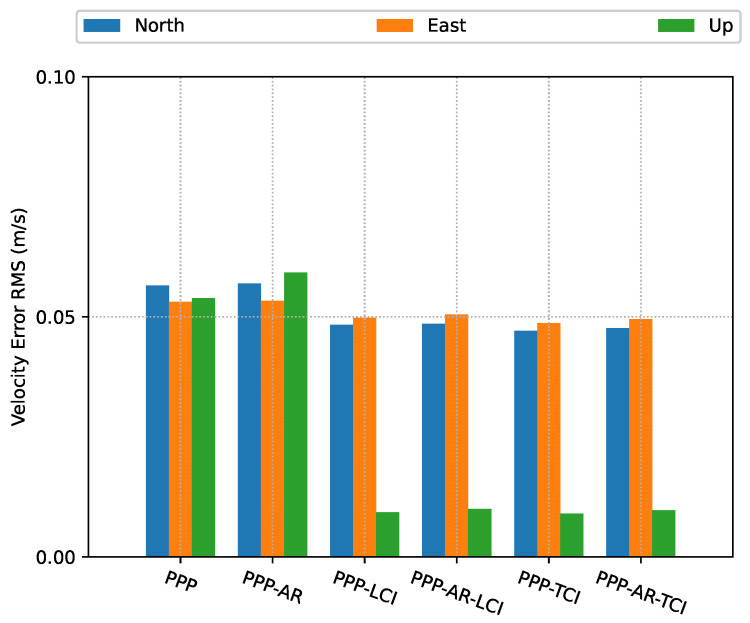
PPP/INS positioning velocity error RMS of the train test on 7 March 2022.

Figure 10 shows the estimated velocity errors. The velocities from the Doppler estimation were much more noisy than that of the integrated solution, especially on the up component. The integrated velocity results had higher accuracy with nearly all components below 5 cm/s, as shown in Figure 11.

**Figure 12:**
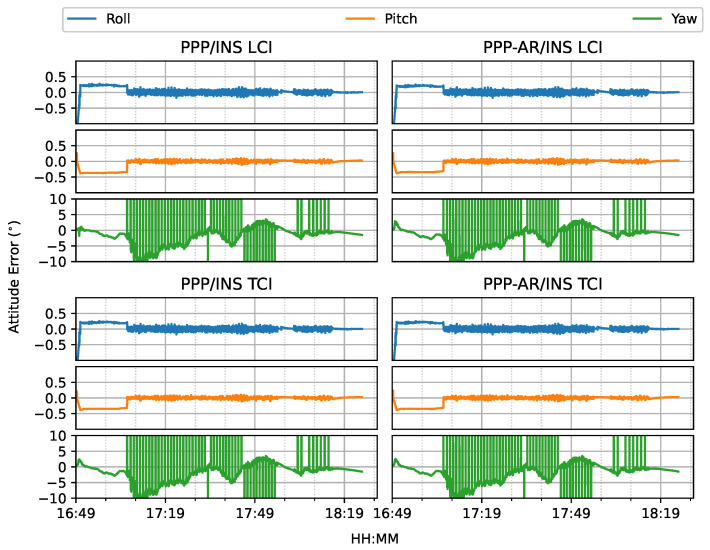
Attitude errors of PPP/INS train positioning test on 7 March 2022.

**Figure 13:**
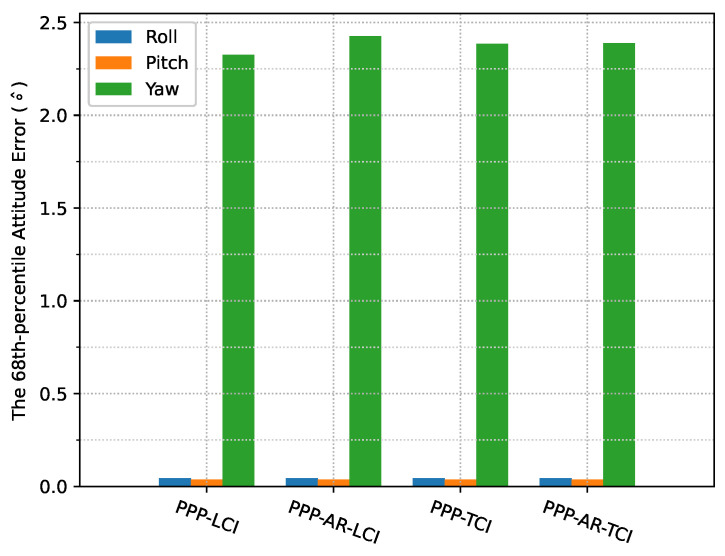
PPP/INS positioning attitude error RMS of the train test on 7 March 2022.

The heading direction in Figure 12 shows frequent spikes because the heading angles of the train included dramatic changes in the movement along the curved path, and the interpolated results were also affected, as compared to the reference. The accurate heading determination required additional alignment for this PPP/INS system. The roll and pitch errors were below 0.1${}^{\circ }$, as shown in Figure 13.

### 3.3. Complex Road Positioning Test Results

#### 3.3.1. Positioning Error Evaluation

**Figure 14:**
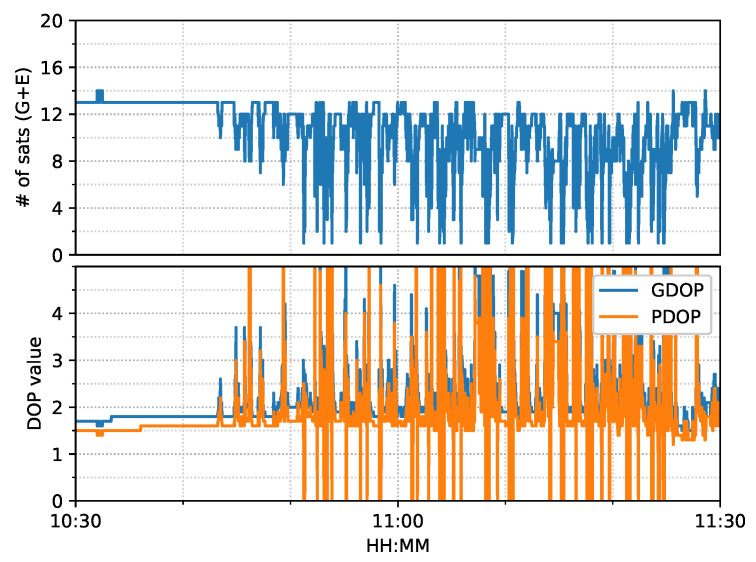
GNSS satellite information of complex road test on 11 February 2022.

**Figure 15:**
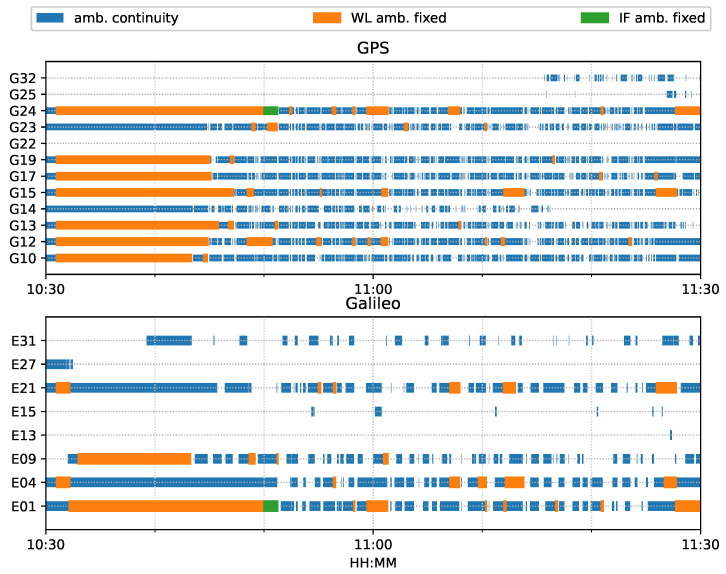
Phase ambiguity status of road bridge test on 11 February 2022.

Figure 14 shows the number of observed satellites had a number of significant drops, and the frequent spikes were in the DOP values in this van test. From the ambiguity status in Figure 15, only G24 and E01 had fixed ionosphere-free (IF) or narrow-lane (NL) ambiguities. These two IF ambiguities were actually fixed as data, but the remaining ambiguities could not be fixed due to the frequent interruptions. Single WL ambiguity resolution (AR) was not beneficial to the solution because the WL ambiguity was estimated from the geometry-free MW combination. As a consequence, the PPP-AR results were equivalent to PPP only with an uncombined bias correction. We used the new notation ’PPP + bias’ to indicate this condition.

**Figure 16:**
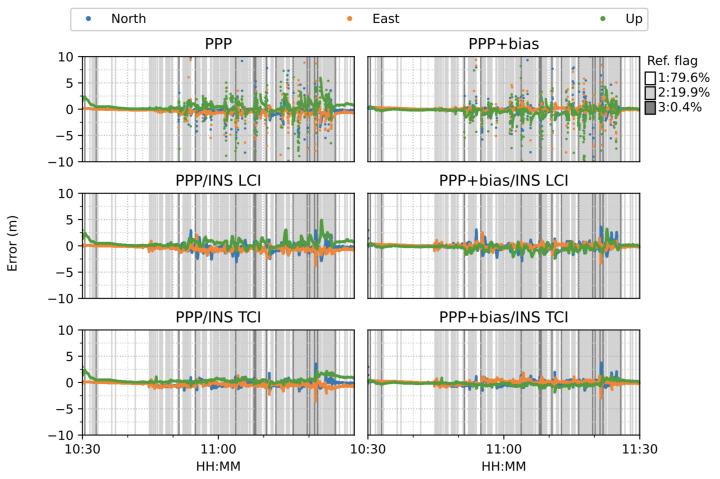
GPS/Galileo PPP/INS results of complex road test on 11 February 2022. Different background color indicates different quality or flag of reference solutions (Ref. flag in the figure). The same below.

Figure 16 shows the six modes of positioning results. The van started moving at around 10:45. A total of 79.6% of the reference Inertial Explorer solutions were ambiguity-fixed. The PPP and PPP + bias solutions suffered from frequent noisy divergence in all directions during the movement but still re-converged rapidly. The solution divergence was caused by a drastic drop of observed satellite numbers and GNSS signal gaps as the van passed through a bridge or a tunnel. The PPP/INS integrated results for both LCI and TCI effectively eliminated the noisy spikes and conserved high accuracy throughout the test. The INS acted as a good complement for the short GNSS signal blockage. Moreover, the TCI solutions achieved the best accuracy. In particular, the additional uncombined bias correction improved the related PPP, LCI, and TCI further, especially for the up component.

**Figure 17:**
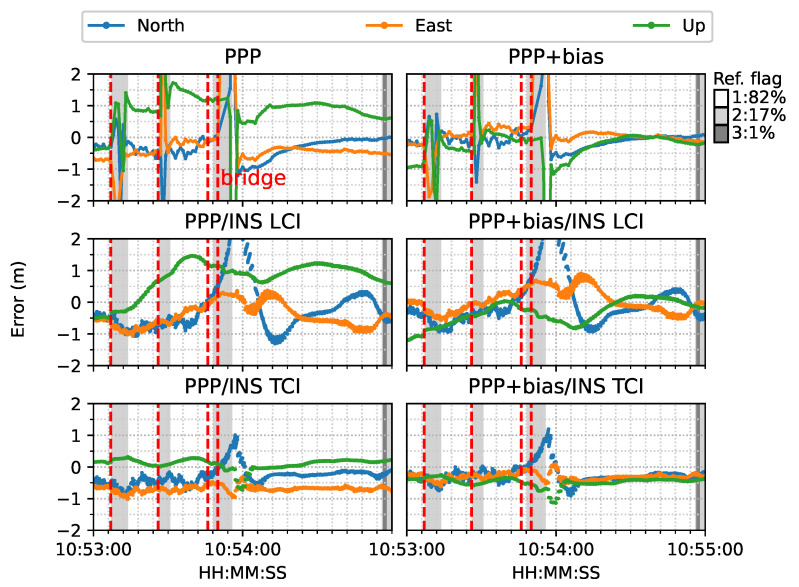
Re-convergence example 1. The red lines indicate the epochs under bridges (The same below).

**Figure 18:**
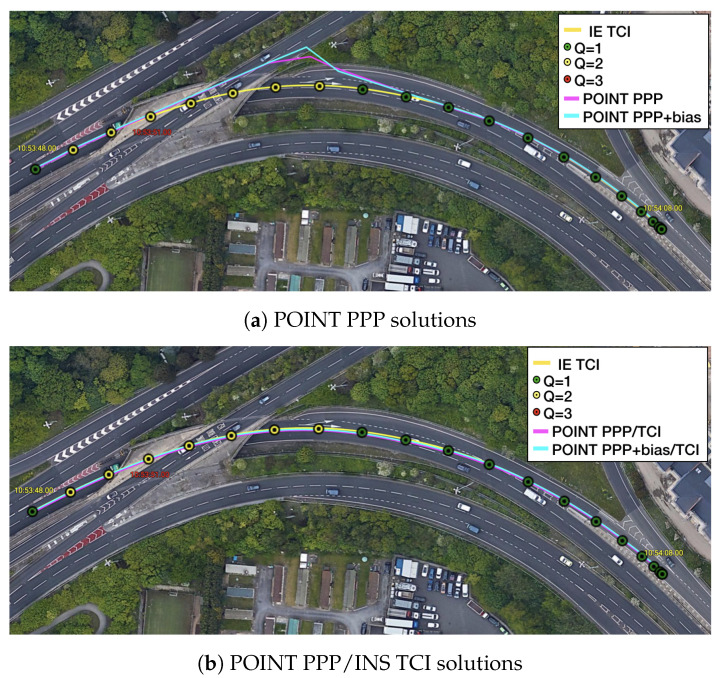
Re-convergence example 1 on Google Earth. The red epoch 10:53:51 signifies when the van entered a tunnel. IE stands for the reference software Inertial Explorer (The same below).

Figure 17 is a two-minute example of the signal outage due to bridges from Figure 16. It was clear that after each bridge epoch (red vertical line), the PPP converged rapidly within a couple of seconds. The LCI and TCI solutions maintained a high precision (decimeter-level) horizontally, and TCI obtained more accurate and steady height solutions. With the real-time uncombined bias correction, all solutions were more aggregated, especially for the height component, and TCI achieved the highest accuracy. Nevertheless, PPP + bias also converged at a comparably high accuracy at the end of the session. It was also shown that at the forth red-line epoch (10:53:50), the TCL and LCI solutions had significant drifts. TCI took around 6 s to recover steady solutions and about 12 s for the LCI to fall below 1 m. The TCI solutions also maintained a higher accuracy of less than 1 m in all directions during the drift.This drift was caused by a 4 s data gap when the van travelled through a tunnel instead of a short bridge, which is illustrated in Figure 18. The PPP solutions drifted severely after entering the tunnel while the TCI solutions were consistent with the reference solutions.

**Figure 19:**
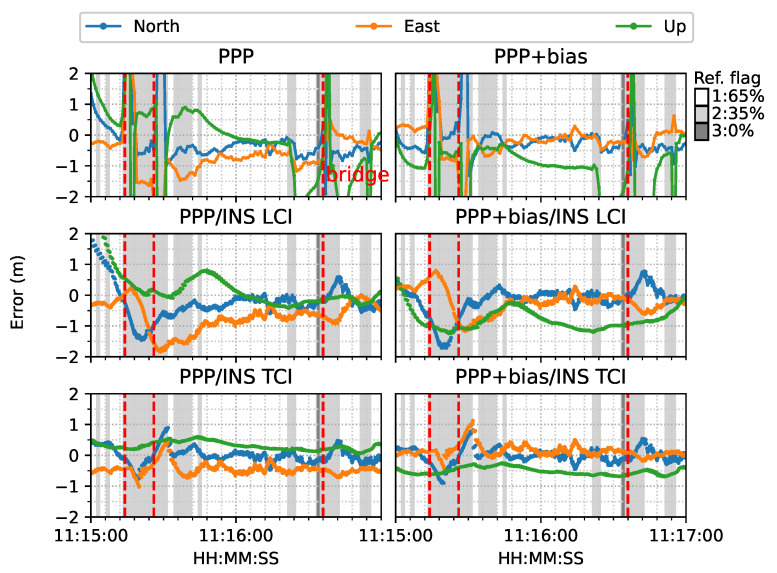
Re-convergence example 2. The red epochs 11:15:14 and 11:15:26 signify that the van was under a bridge.

**Figure 20:**
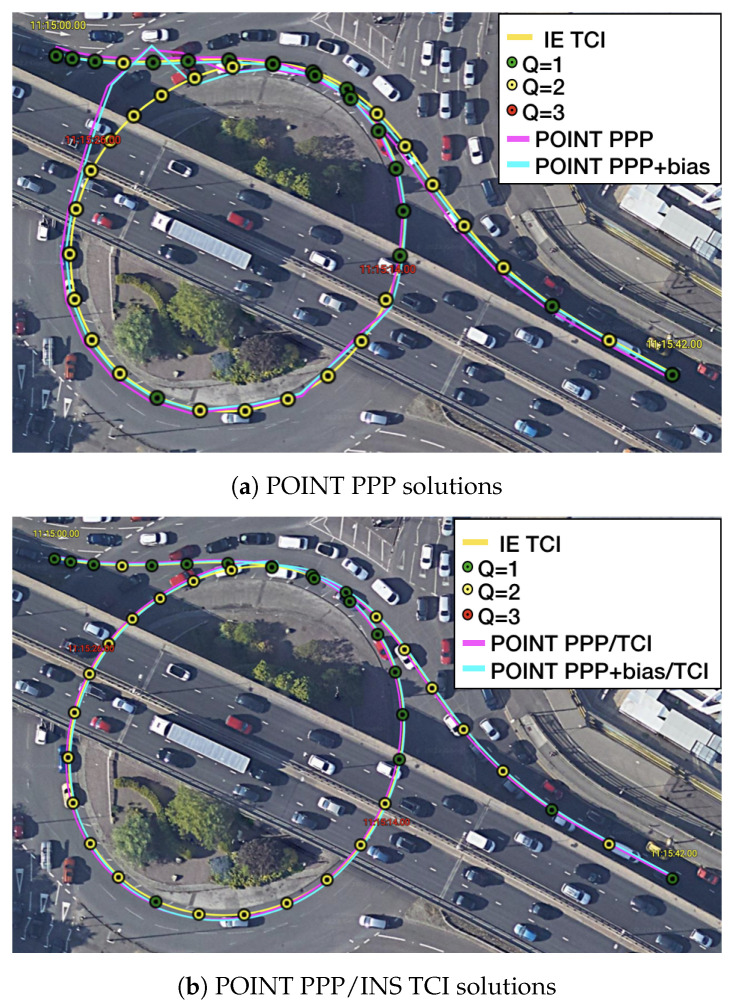
Re-convergence example 2 on Google Earth.

Figure 19 provides another example of the PPP re-convergence after the bridge epochs. In this case, the additional uncombined bias correction accelerated the PPP convergence to a few seconds horizontally and also improved the PPP, LCI, and TCI positioning accuracy. TCI, as expected, achieved the best performance and preserved a decimeter-level accuracy during the session. Figure 20 is the related satellite view of the first two bridge epochs. As in Figure 18, the PPP-only solutions showed large drifts while under a bridge at 11:15:26 while the TCI solutions provided the best consistency with the reference.

**Figure 21:**
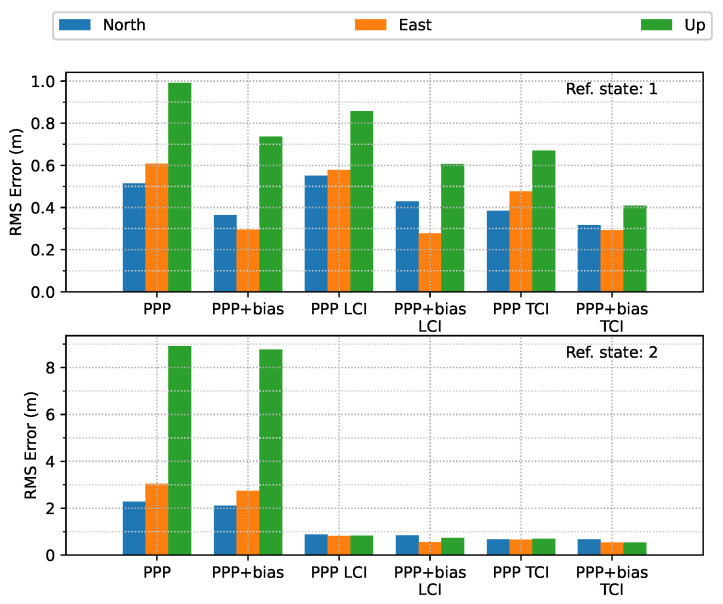
Error RMS of real-time GPS/Galileo PPP/INS results of road bridge test on 11 February 2022.

Figure 21 shows the overall error RMS of each positioning mode. The comparison was divided by the quality of the reference solutions, e.g., 1 and 2. The quality or state 1 solution was the majority, as shown in Figure 16, and usually corresponded to epochs with good satellite visibility. The quality 2 reference solution usually had poor observation conditions, e.g., under bridges. For quality 1, without bias correction, TCI achieved the best accuracy in the north (N), east (E), and up (U) directions, at 38, 48 and 67 cm, respectively, and had an improvement of 25%, 21%, and 32%, respectively, as compared to the PPP results of 51, 61, and 99 cm, respectively.After applying the uncombined biases to PPP, LCI, and TCI, a higher positioning accuracy was obtained, as compared to the uncorrected solutions. TCI still offered the highest accuracy of 32, 29, and 41 cm for the N/E/U component, respectively, which was improved by 18%, 40%, and 39%, respectively, as compared to uncorrected TCI and by 37%, 52%, and 59% with PPP-only solutions, respectively. The east accuracy after bias correction was almost identical for the three modes, and the up component was the most improved. The LCI performance was in between that of PPP and TCI. For quality 2, the integrated solutions had a substantial improvement, especially for the height component, which was 94%. The horizontal accuracy was improved from around 2.5 m to a decimeter level. LCI and TCI had comparable accuracy, which bias correction improved further.

#### 3.3.2. Velocity and Attitude Error Evaluation

**Figure 22:**
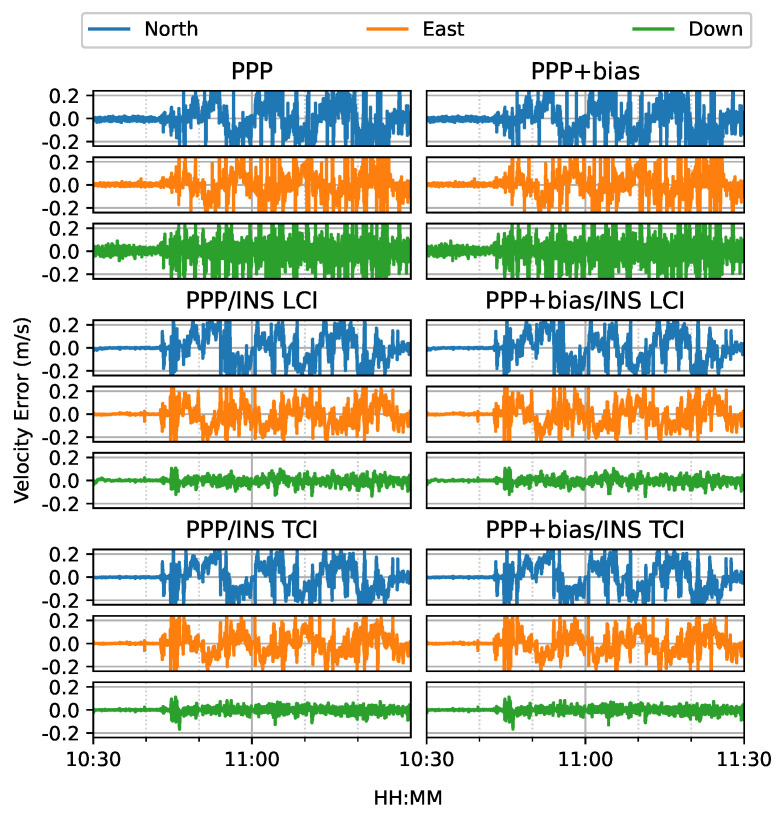
GPS/Galileo PPP/INS velocity results of road bridge test on 11 February 2022 with respect to IE RTK/INS TCI solutions.

**Figure 23:**
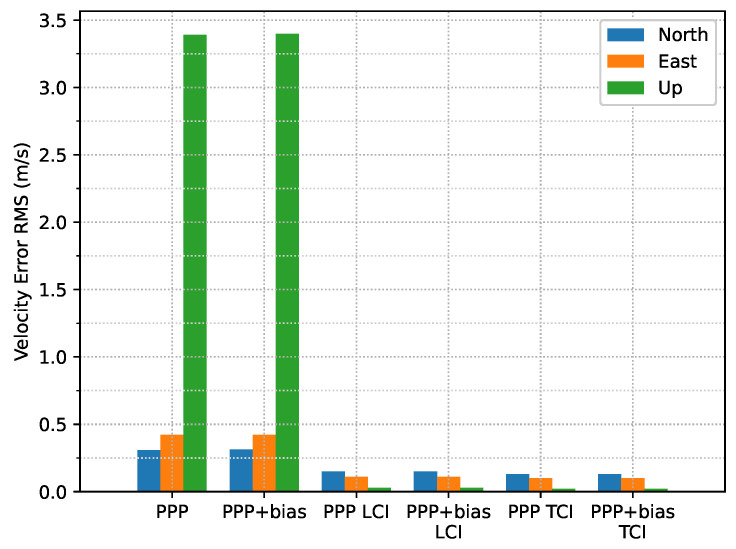
Velocity error RMS of real-time GPS/Galileo PPP/INS results of road bridge test on 11 February 2022.

Figure 22 shows the estimated velocity errors of the GNSS antenna. It was clear that the integrated solutions were more aggregated than the Doppler estimation. The effect of the bias correction was not significant. As shown in Figure 23, the PPP or Doppler velocity accuracy was 0.31, 0.42, and 3.39 m/s in the N/E/U directions, respectively, and 0.15, 0.11, and 0.03 m/s for that of LCI, respectively. The TCI results were nearly identical to the LCI. The integrated solution achieved an improvement of 52%, 74%, and 99%, respectively.

**Figure 24:**
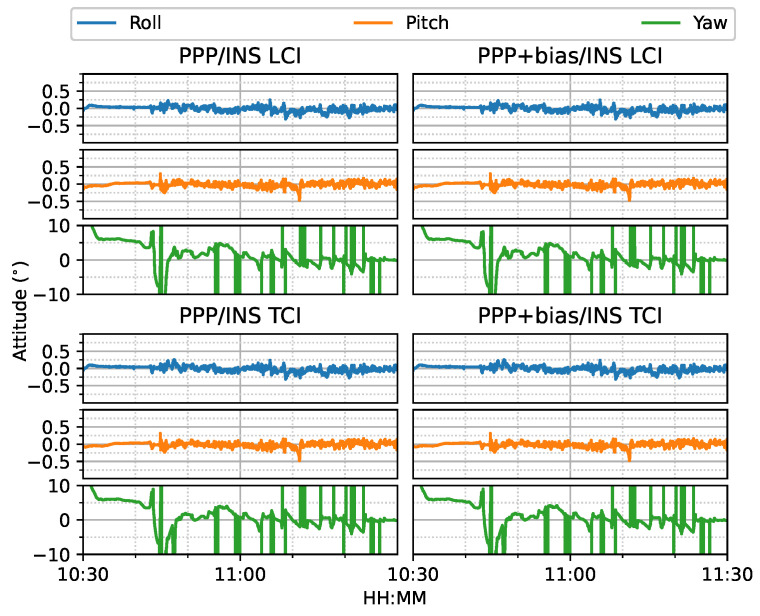
GPS/Galileo PPP/INS attitude results of road bridge test on 11 February 2022 with respect to IE RTK/INS TCI solutions.

**Figure 25:**
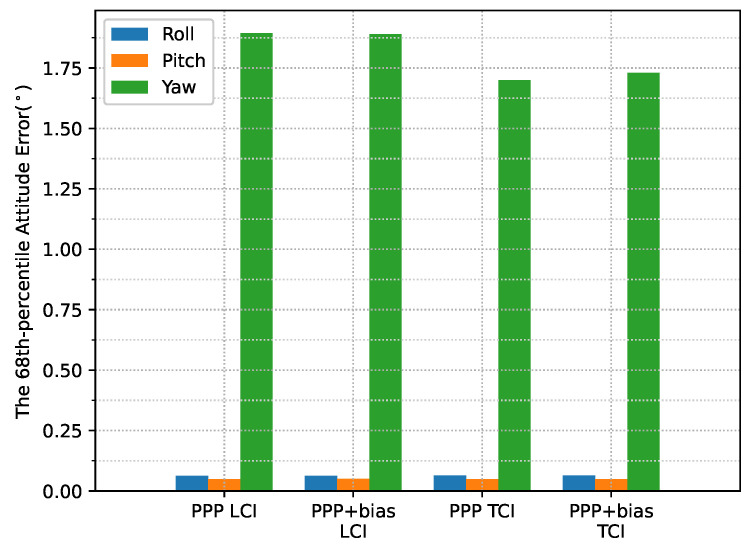
Attitude error RMS of real-time GPS/Galileo PPP/INS results of road bridge test on 11 February 2022.

Figure 24 shows the attitude errors. The yaw (heading) errors had many spikes, as shown in Figure 12, corresponding to dramatic change of heading during the movement. As shown in Figure 25, the LCI attitude accuracy was 0.06, 0.05, and 1.89${}^{\circ }$ for roll, pitch, and yaw angles, respectively. The TCI attitudes had an improvement of 10% in the heading accuracy. The effect of the bias correction was still negligible.

### 3.4. City Center Positioning Test Results

#### 3.4.1. Positioning Error Evaluation

**Figure 26:**
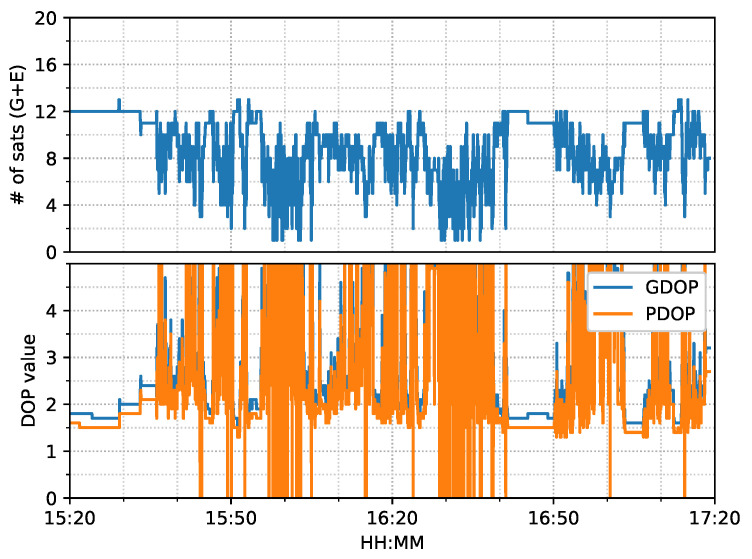
GNSS satellite information of city center test on 27 January 2022.

**Figure 27:**
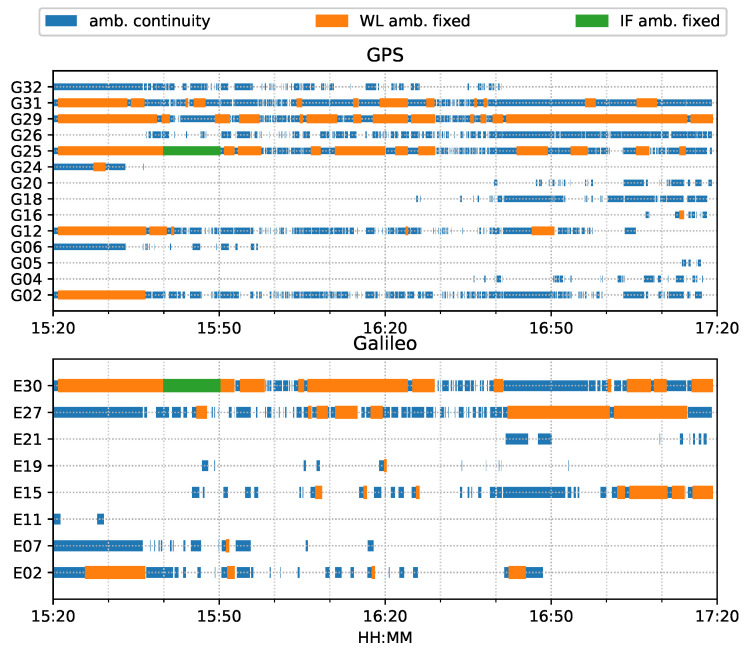
Phase ambiguity status of city center test on 27 January 2022.

Figure 26 shows that the number of tracked satellites in the city center test had a significant decrease at around 16:00 and 16:30 and required more time to recover peak values, as compared to Figure 14, which indicated harsh signal reception in the city center. Due to vegetation and high-rise canyons, GNSS signal blockage or gapes frequently occurred during the city test. As in Figure 27, the ambiguities of the tracked satellites were not consecutive and had frequent and long interruptions, which made it difficult to fix the IF or NL ambiguities, as shown in Figure 15.

**Figure 28:**
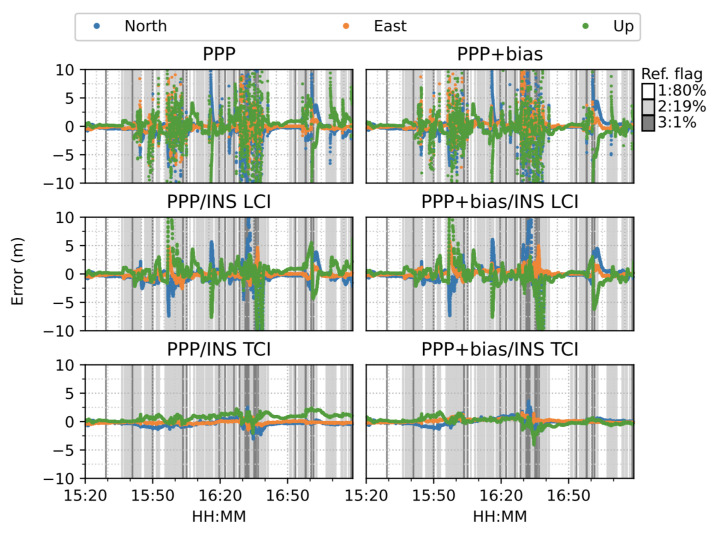
Real-time GPS/Galileo PPP/INS results of city center test on 27 January 2022 with respect to IE RTK/INS TCI solutions.

The van started moving at around 15:35 and stopped for about 15 min from around 16:35. As shown in Figure 28, the PPP solutions re-converged significantly several times. PPP + bias improved the results to some degree, but the frequent re-convergence continued to be an issue.The LCI solution mitigated the noisy spikes of the PPP, but the improvement were marginal. The bias correction added more aggregation in the up component. The TCI solutions, however, eliminated the divergence effectively and obtained the best performance during the test. TCI with bias correction improved the results further, especially for the up component.

**Figure 29:**
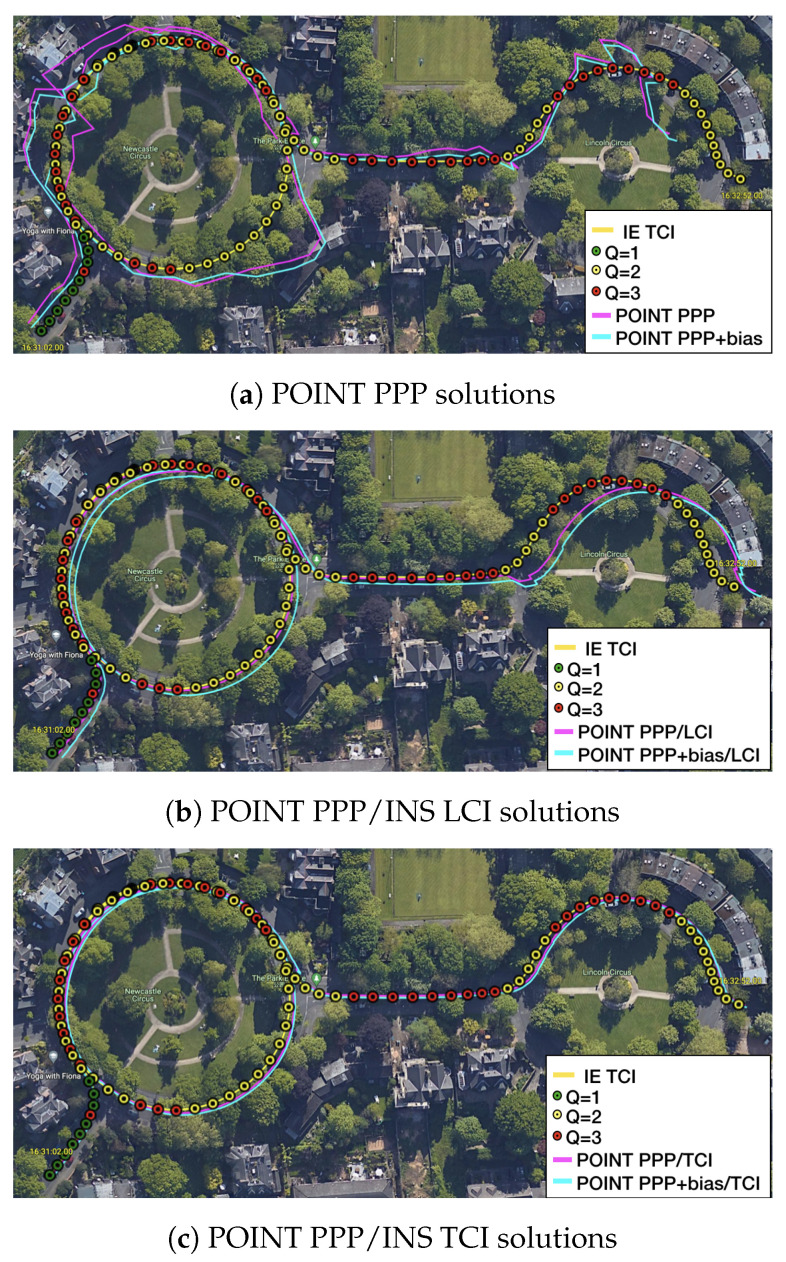
Example 1 of city center test on Google Earth.

Figure 29 is an example of satellite view from 16:31:02 to 16:32:52. Vegetation was clustered along the curved path, and the reference solutions showed a lot of red states, indicating a challenging signal reception environment. As expected, the PPP solutions as well as as the bias corrected solutions had substantial inconsistencies along the path. The LCI solutions were better, yet they still had a clear shift from the reference. The TCI solutions again provided the best consistency.

**Figure 30:**
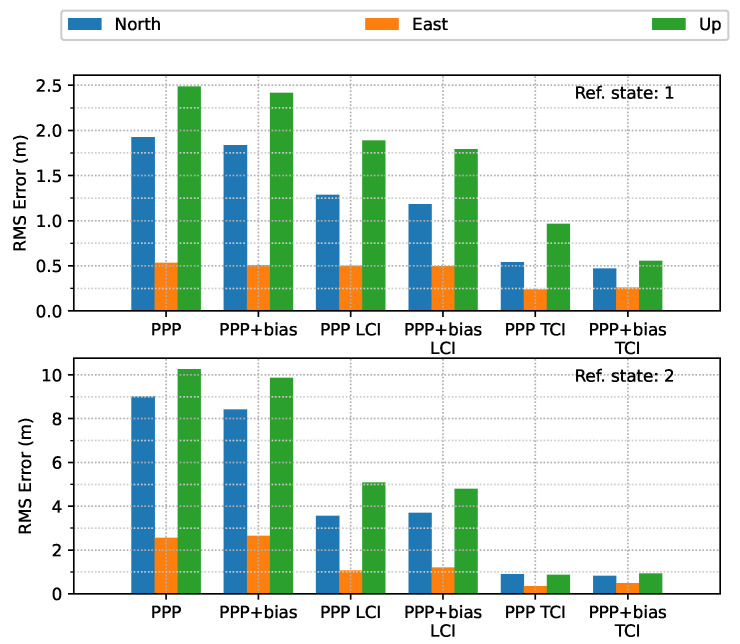
Error RMS of real-time GPS/Galileo PPP/INS results of city center test on 27 January 2022 with respect to IE RTK/INS TCI solutions.

In terms of overall error RMS, as shown in Figure 30, the contribution of the bias correction was mainly for the up component, and the horizontal accuracy was almost equivalent to the solutions without biases for PPP, LCI, and TCI. With respect to the reference state or quality 1 solutions, TCI after bias correction achieved the best performance with an accuracy of 47, 26, and 55 cm in the N/E/U directions, respectively.

#### 3.4.2. Velocity and Attitude Errors

**Figure 31:**
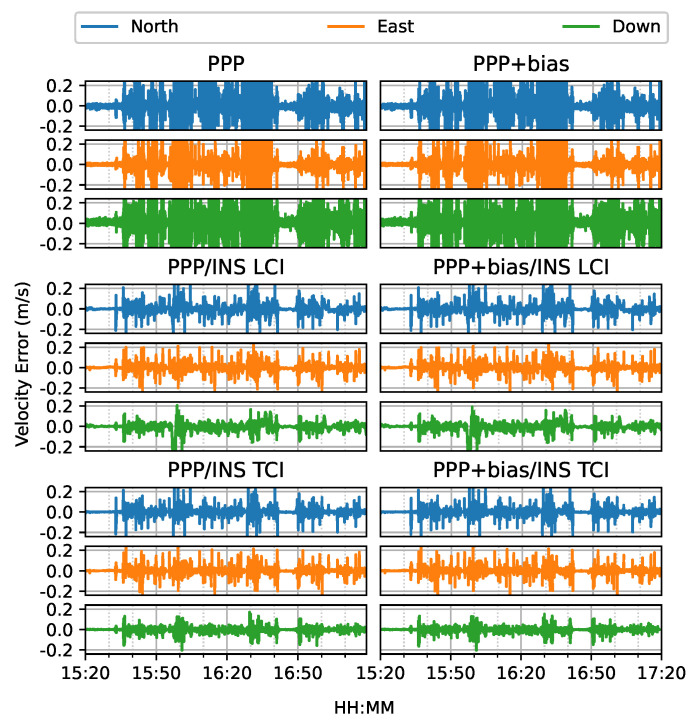
Real-time GPS/Galileo PPP/INS velocity errors of city center test on 27 January 2022 with respect to IE RTK/INS TCI solutions.

**Figure 32:**
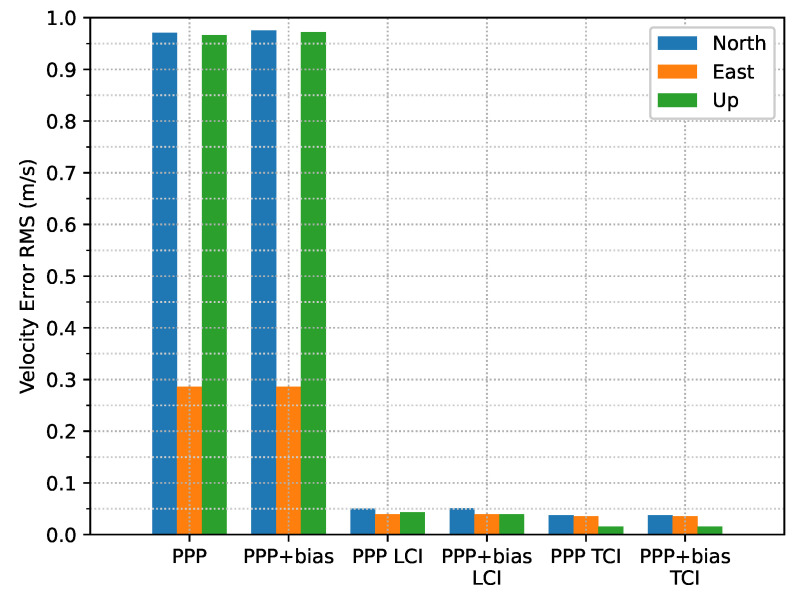
Velocity error RMS of real-time GPS/Galileo PPP/INS positioning of city center test on 27 January 2022 with respect to IE RTK/INS TCI solutions.

From Figure 31 and Figure 32, the integrated velocity solutions have higher accuracy than Doppler estimation in all directions as in Figure 22 and Figure 23.

**Figure 33:**
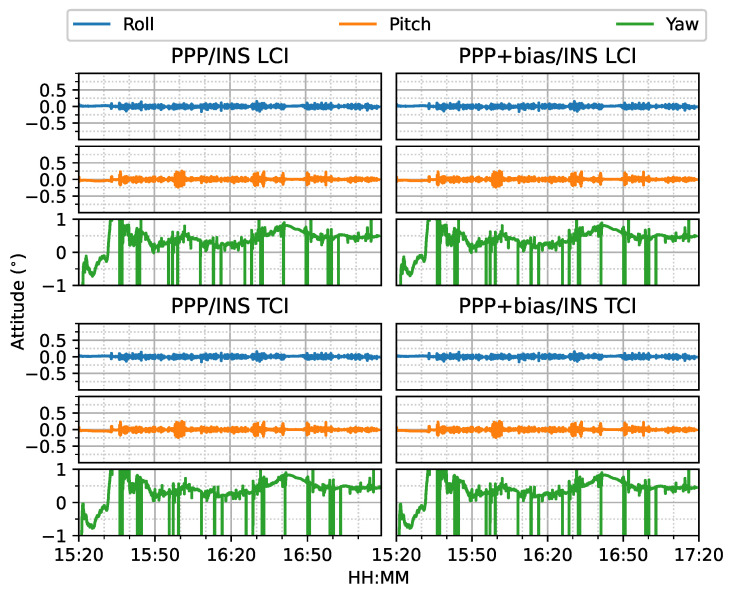
Real-time GPS/Galileo PPP/INS attitude errors of city center test on 27 January 2022 with respect to IE RTK/INS TCI solutions.

**Figure 34:**
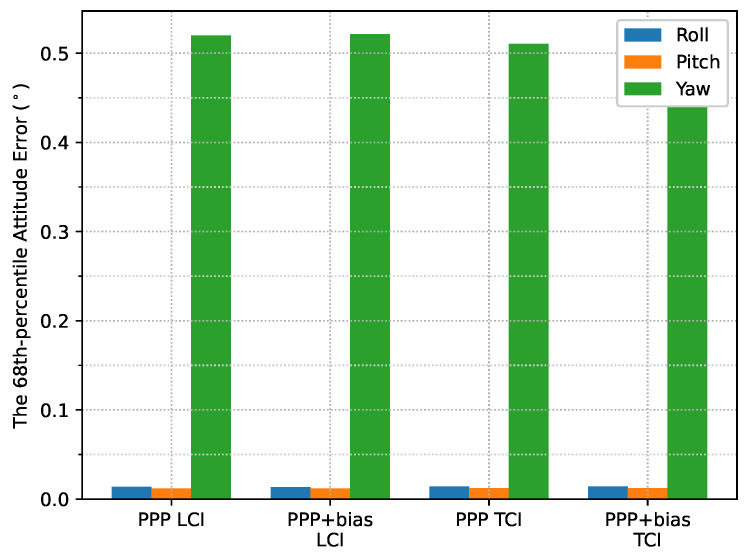
Attitude error RMS of real-time GPS/Galileo PPP/INS positioning of city center test on 27 January 2022 with respect to IE RTK/INS TCI solutions.

The attitude heading errors also show several spikes in Figure 33, which is similar to Figure 12 and Figure 24. The LCI and TCI attitude solutions are almost identical from Figure 34.

## 4. Discussion

The dramatic change in heading increased the difficulty of precise yaw angle determination in this PPP/INS integrated system. External heading alignment was required to improve this defect. The GPS/Galileo multi-frequency IF PPP model using uncombined bias products should be further evaluated, as the current dual-frequency model may still require more than 15 min to converge to high accuracy. The TCI of the PPP and the tactical-grade IMU preserved the decimeter-level accuracy during the short signal intervals, but the IF PPP integration with the low-cost IMU requires further investigation.

## 5. Conclusions

In this study, we evaluated the GPS/Galileo PPP/INS positioning performance with CNES real-time orbit, clock, and uncombined bias products through a train positioning test in an open sky environment and a van positioning test on a complex road with bridges and tunnels. The uncombined bias products were used for the resolution of dual-frequency ionosphere-free ambiguity.

In the train test, it was found that ambiguity-float PPP had an almost identical performance with either LCI or TCI and reached an accuracy of 8.5, 5.7, and 4.9 cm in the north (N), east(E) and up (U)directions, respectively. After the phase ambiguity resolution (AR), significant improvements on the east error component were achieved, which were 47%, 40%, and 38% for PPP-AR, PPP-AR/INS LCI, and PPP-AR/INS TCI, respectively. Under good observational conditions, the benefit of the additional inertial measurements for the positioning results was marginal. However, for the van tests in the complex road and city center, frequent drops of a number of visible satellites directly caused multiple re-convergences in the PPP solutions, in which the IF AR was not feasible to obtain, though the phase biases were corrected. The TCI achieved most accurate results, which were 32, 29, and 41 cm for the N/E/U components, respectively, and also effectively eliminated the solution re-convergence in PPP.

## Figures and Tables

**Table 1:** Sensor sampling rates, lever arm, and installation angles from the two tests.

	Train Test		Van Test
GNSS data rate	10 Hz		1 Hz
IMU data rate		200 Hz	
Installation angle	180${}^{\circ }$ (roll), 0${}^{\circ }$ (pitch), 0${}^{\circ }$ (yaw)		from IMU frame to vehicle frame
Lever arm	0.783 m, 0.156 m, −1.011 m		−0.626 m, 0.307 m, −0.543 m

**Table 2:** POINT PPP settings.

Constellation	GPS & Galileo
Frequency	L1/L2 E1/E5a
Meas. noise	Code: 0.2 m; Phase: 0.01 cycle
Parameter estimation	Extended Kalman Filter
Orbit and clock	CNES real-time products
Biases	CNES real-time uncombined bias products
Ambiguity resolution	Bootstrapping
Elevation cut-off	10${}^{\circ }$
Weighting function	$\frac{1.001}{\sqrt{0.002001+sin^{2}\theta }}$ where θ is the elevation angle (radian)
Antenna PCO/PCV correction	igs14_2188.atx
	Earth orientation parameters: IERS EOP 14 C04
	(IAU2000A); Solar system body ephemerides:
	NASA NAIF SPICE files
Phase windup	[40]
Phase cycle slip detection	[48]
Troposphere	Saastamoinen model for the hydrostatic delay
	Niell mapping function
	Estimation on the zenith wet delay
	Initial variance: 0.5 m; Model noise: 0.005 mm/s
Ionosphere	Higher-order terms are ignored
	Ionosphere-free combination (see Equations (4) and (7))
Receiver clock offset	Estimated as white noise; Model noise 1000 m/s
Receiver state	Model noise: 10 m/s for X Y Z

## Data Availability

The CNES real-time satellite orbit, clock, and uncombined bias products can be freely downloaded from: http://www.ppp-wizard.net/daily.html (accessed on 17 February 2023).

## Abbreviations

The following abbreviations are used in this manuscript:

GNSS	Global Navigation Satellite System
INS	Inertial Navigation System
PPP	Precise Point Positioning
IFCB	Inter Frequency Clock Bias
RTCM	Radio Technical Commission for Maritime Services
WL	Widelane
NL	Narrowlane
AR	Ambiguity Resolution
MW	Melbourne–Wübbena
IF	Ionosphere-free
LCI	Loosely coupled Integration
TCI	Tightly coupled Integration

## References

[1] Zumberge J., Heflin M., Jefferson D., Watkins M., Webb F. (1997). Precise Point Positioning for the Efficient Furthermore, Robust Analysis of GPS Data from Large Networks. J. Geophys. Res..

[2] Bisnath S., Gao Y. (2008). Current State of Precise Point Positioning and Future Prospects and Limitations. Observing Our Changing Earth.

[3] Ge M., Gendt G., Rothacher M., Shi C., Liu J. (2008). Resolution of GPS carrier-phase ambiguities in Precise Point Positioning (PPP) with daily observations. J. Geod..

[4] Laurichesse D., Mercier F., Berthias J.P., Broca P., Cerri L. (2009). Integer ambiguity resolution on undifferenced GPS phase measurements and its application to PPP and satellite precise orbit determination. Navig. J. Inst. Navig..

[5] Collins P., Bisnath S., Lahaye F., Héroux P. (2010). Undifferenced GPS Ambiguity Resolution Using the Decoupled Clock Model and Ambiguity Datum Fixing. Navigation.

[6] Tegedor J., Liu X., Jong K., Goode M., Ovstedal O., Vigen E. Estimation of Galileo Uncalibrated Hardware Delays for Ambiguity Fixed Precise Point Positioning. Proceedings of the 27th International Technical Meeting of the Satellite Division of The Institute of Navigation (ION GNSS+ 2014).

[7] Banville S., Resources N., Banville S. (2016). GLONASS ionosphere-free ambiguity resolution for precise point positioning. J. Geod..

[8] Pan L., Zhang X., Li X., Liu J., Li X. (2017). Characteristics of inter-frequency clock bias for Block IIF satellites and its effect on triple-frequency GPS precise point positioning. GPS Solut..

[9] Li X., Li X., Yuan Y., Zhang K., Zhang X., Wickert J. (2018). Multi-GNSS phase delay estimation and PPP ambiguity resolution: GPS, BDS, GLONASS, Galileo. J. Geod..

[10] Montenbruck O., Hugentobler U., Dach R., Steigenberger P., Hauschild A. (2012). Apparent clock variations of the Block IIF-1 (SVN62) GPS satellite. GPS Solut..

[11] Pan L., Zhang X., Guo F., Liu J. (2019). GPS inter-frequency clock bias estimation for both uncombined and ionospheric-free combined triple-frequency precise point positioning. J. Geod..

[12] Guo J., Geng J. (2018). GPS satellite clock determination in case of inter-frequency clock biases for triple-frequency precise point positioning. J. Geod..

[13] Li P., Jiang X., Zhang X., Ge M., Schuh H. (2020). GPS + Galileo + BeiDou precise point positioning with triple-frequency ambiguity resolution. GPS Solut..

[14] Geng J., Guo J., Meng X., Gao K. (2020). Speeding up PPP ambiguity resolution using triple-frequency GPS/BeiDou/Galileo/QZSS data. J. Geod..

[15] Li X., Li X., Liu G., Feng G., Yuan Y., Zhang K., Ren X. (2019). Triple-frequency PPP ambiguity resolution with multi-constellation GNSS: BDS and Galileo. J. Geod..

[16] Li X., Liu G., Li X., Zhou F., Feng G., Yuan Y., Zhang K. (2020). Galileo PPP rapid ambiguity resolution with five-frequency observations. GPS Solut..

[17] Laurichesse D. Phase Biases Estimation for Integer Ambiguity Resolution. 2012. https://igs.bkg.bund.de/root_ftp/NTRIP/documentation/PPP-RTK2012/14_Laurichesse_Denis.pdf.

[18] Laurichesse D., Langley R. Handling the Biases for Improved Triple-Frequency PPP Convergence. *GPS World*. 2015. https://www.gpsworld.com/innovation-carrier-phase-ambiguity-resolution/.

[19] Laurichesse D. Phase Biases for Ambiguity Resolution from an Undifferenced to an Uncombined Formulation. 2014. http://www.ppp-wizard.net/Articles/WhitePaperL5.pdf.

[20] Laurichesse D., Banville S. Innovation: Instantaneous centimeter-level multi-frequency precise point positioning. *GPS World*. 2018. https://www.gpsworld.com/innovation-instantaneous-centimeter-level-multi-frequency-precise-point-positioning/.

[21] Geng J., Guo J. (2020). Beyond three frequencies: An extendable model for single-epoch decimeter-level point positioning by exploiting Galileo and BeiDou-3 signals. J. Geod..

[22] Geng J., Wen Q., Zhang Q., Li G., Zhang K. (2022). GNSS observable-specific phase biases for all-frequency PPP ambiguity resolution. J. Geod..

[23] Li X., Li X., Jiang Z., Xia C., Shen Z., Wu J. (2022). A unified model of GNSS phase/code bias calibration for PPP ambiguity resolution with GPS, BDS, Galileo and GLONASS multi-frequency observations. GPS Solut..

[24] Scherzinger B.M. Precise robust positioning with inertial/GPS RTK. Proceedings of the 13th International Technical Meeting of the Satellite Division of The Institute of Navigation (ION GPS 2000).

[25] Shin E.H. (2001). Accuracy Improvement of Low Cost INS/GPS for Land Applications. Master’s Thesis.

[26] Zhang Y., Gao Y. (2008). Integration of INS and un-differenced GPS measurements for precise position and attitude determination. J. Navig..

[27] Héroux P., Kouba J. (2001). GPS precise point positioning using IGS orbit products. Phys. Chem. Earth Part A Solid Earth Geod..

[28] Kouba J. (2009). A Guide to using international GNSS Service ( IGS ) Products. Geod. Surv. Div. Nat. Resour. Can. Ott..

[29] Abd Rabbou M., El-Rabbany A. (2015). Tightly coupled integration of GPS precise point positioning and MEMS-based inertial systems. GPS Solut..

[30] Gao Z., Ge M., Shen W., Li Y., Chen Q., Zhang H., Niu X. (2017). Evaluation on the impact of IMU grades on BDS + GPS PPP/INS tightly coupled integration. Adv. Space Res..

[31] Vana S., Naciri N., Bisnath S. Low-cost, dual-frequency PPP GNSS and MEMS-IMU integration performance in obstructed environments. Proceedings of the 32nd International Technical Meeting of the Satellite Division of the Institute of Navigation, ION GNSS+ 2019.

[32] Han H., Xu T., Wang J. (2016). Tightly coupled integration of GPS ambiguity fixed precise point positioning and MEMS-INS through a troposphere-constrained adaptive kalman filter. Sensors.

[33] Liu S., Sun F., Zhang L., Li W., Zhu X. (2016). Tight integration of ambiguity-fixed PPP and INS: Model description and initial results. GPS Solut..

[34] Zhang X., Zhu F., Zhang Y., Mohamed F., Zhou W. (2019). The improvement in integer ambiguity resolution with INS aiding for kinematic precise point positioning. J. Geod..

[35] Gu S., Dai C., Fang W., Zheng F., Wang Y., Zhang Q., Lou Y., Niu X. (2021). Multi-GNSS PPP/INS tightly coupled integration with atmospheric augmentation and its application in urban vehicle navigation. J. Geod..

[36] Li X., Li X., Huang J., Shen Z., Wang B., Yuan Y., Zhang K. (2021). Improving PPP–RTK in urban environment by tightly coupled integration of GNSS and INS. J. Geod..

[37] Li X., Li X., Li S., Zhou Y., Sun M., Xu Q., Xu Z. (2022). Centimeter-accurate vehicle navigation in urban environments with a tightly integrated PPP-RTK/MEMS/vision system. GPS Solut..

[38] Gu S., Dai C., Mao F., Fang W. (2022). Integration of Multi-GNSS PPP-RTK/INS/Vision with a Cascading Kalman Filter for Vehicle Navigation in Urban Areas. Remote Sens..

[39] Laurichesse D., Privat A. An open-source PPP client implementation for the CNES PPP-WIZARD demonstrator. Proceedings of the 28th International Technical Meeting of the Satellite Division of the Institute of Navigation, ION GNSS 2015.

[40] Wu J.T., Wu S.C., Hajj G.A., Bertiger W.I., Lichten S.M. (1993). Effects of antenna orientation on GPS carrier phase. Manuscripta Geod..

[41] Melbourne W. The Case for Ranging in GPS-based Geodetic Systems. Proceedings of the 1st International Symposium on Precise Positioning with the Global Positioning System.

[42] Wübbena G. Software developments for geodetic positioning with GPS using TI-4100 code and carrier measurements. Proceedings of the 1st International Symposium on Precise Positioning with the Global Positioning System.

[43] Groves P.D. (2013). Principles of GNSS, Inertial, and Multisensor Integrated Navigation Systems/Paul D. Groves.

[44] Novatel-SPAN-UIMU-LCI Tactical Grade, Low Noise IMU Delivers 3D Position, Velocity and Attitude Solution as Part of SPAN Technology. 2014. http://www.canalgeomatics.com/product_files/novatel-uimu-lci-datasheet_372.pdf.

[45] Leica-Geosystems-AG Leica GS10/GS15 User Manual. 2016. http://www.surveyteq.com/uploads/p_C9C59E1C-40F3-A040-B287-D30E0C6B00A4-1517301551.pdf.

[46] Hide C., Pinchin J., Park D. Development of a low cost multiple GPS antenna attitude system. Proceedings of the 20th International Technical Meeting of the Satellite Division of The Institute of Navigation 2007 ION GNSS 2007.

[47] Zhao L., Blunt P., Yang L. (2022). Performance Analysis of Zero-Difference GPS L1/L2/L5 and Galileo E1/E5a/E5b/E6 Point Positioning Using CNES Uncombined Bias Products. Remote Sens..

[48] Liu Z. (2011). A new automated cycle slip detection and repair method for a single dual-frequency GPS receiver. J. Geod..

[49] NovAtel Inertial Explorer Show Map Window, Waypoint User Documentation. 2022. https://docs.novatel.com/Waypoint/Content/GrafNav/Show_Map_Window.htm.

[50] Takasu T. RTKLIB: Open Source Program Package for RTK-GPS. 2009. http://gpspp.sakura.ne.jp/paper2005/foss4g_2009_rtklib.pdf.

